# Checklist of the mosquito fauna (Diptera, Culicidae) of Cambodia

**DOI:** 10.1051/parasite/2021056

**Published:** 2021-08-10

**Authors:** Pierre-Olivier Maquart, Didier Fontenille, Nil Rahola, Sony Yean, Sébastien Boyer

**Affiliations:** 1 Medical and Veterinary Entomology Unit, Institut Pasteur du Cambodge 5 BP 983 Blvd. Monivong 12201 Phnom Penh Cambodia; 2 MIVEGEC, University of Montpellier, CNRS, IRD 911 Avenue Agropolis 34394 Montpellier France

**Keywords:** Taxonomy, Mosquito, Biodiversity, Vectors, Medical entomology, Asia

## Abstract

Between 2016 and 2020, the Medical and Veterinary Entomology unit of the *Institut Pasteur du Cambodge* collected over 230,000 mosquitoes. Based on this sampling effort, a checklist of 290 mosquito species in Cambodia is presented. This is the first attempt to list the Culicidae fauna of the country. We report 49 species for the first time in Cambodia. The 290 species belong to 20 genera: *Aedeomyia* (1 sp.), *Aedes* (55 spp.), *Anopheles* (53 spp.), *Armigeres* (26 spp.), *Coquillettidia* (3 spp.), *Culex* (57 spp.), *Culiseta* (1 sp.), *Ficalbia* (1 sp.), *Heizmannia* (10 spp.), *Hodgesia* (3 spp.), *Lutzia* (3 spp.), *Malaya* (2 spp.), *Mansonia* (5 spp.), *Mimomyia* (7 spp.), *Orthopodomyia* (3 spp.), *Topomyia* (4 spp.), *Toxorhynchites* (4 spp.), *Tripteroides* (6 spp.), *Uranotaenia* (27 spp.), and *Verrallina* (19 spp.). The Cambodian Culicidae fauna is discussed in its Southeast Asian context. Forty-three species are reported to be of medical importance, and are involved in the transmission of pathogens.

## Abstract (fr)

Entre 2016 et 2020, plus de 230 000 moustiques ont été capturés par l’Unité d’Entomologie Médicale et Vétérinaire de l’Institut Pasteur du Cambodge. Sur la base de ces collectes, une liste des 290 espèces de moustiques présentes au Cambodge est présentée. Il s’agit de la première tentative de répertorier la faune Culicidae du pays. Quarante-neuf espèces sont signalées pour la première fois au Cambodge. Les 290 espèces appartiennent à vingt genres : *Aedeomyia* (1 sp.), *Aedes* (55 spp.), *Anopheles* (53 spp.), *Armigeres* (26 spp.), *Coquillettidia* (3 spp.), *Culex* (57 spp.), *Culiseta* (1 sp.), *Ficalbia* (1 sp.), *Heizmannia* (10 spp.), *Hodgesia* (3 spp.), *Lutzia* (3 spp.), *Malaya* (2 spp.), *Mansonia* (5 spp.), *Mimomyia* (7 spp.), *Orthopodomyia* (3 spp.), *Topomyia* (4 spp.), *Toxorhynchites* (4 spp.), *Tripteroides* (6 spp.), *Uranotaenia* (27 spp.) et *Verrallina* (19 spp.). La faune des Culicidés cambodgiens est discutée dans son contexte sud-est asiatique. Quarante-trois espèces sont d’importance médicale et impliquées dans la transmission de pathogènes.

## Introduction

The Greater Mekong Sub-region (GMS) is undergoing unprecedented demographic and environmental changes that threaten its ecosystem stability [109]. The sub-region is composed of six countries: China (Yunnan province and Guangxi Zhuang autonomous region), Laos, Myanmar, Thailand, Vietnam and Cambodia. Cambodia, bordered by Thailand, Laos and Vietnam, is considered the lowland region of the GMS. The country hosts the largest freshwater lake in Southeast Asia: the Tonle Sap, characterised by an unusual hydrological regime. Due to the biannual flow reversal of the Tonle Sap river into the Mekong river, the lake can become four times larger in the rainy season than during the dry season. When flooded, the lake covers up to three million hectares, making it one of the world’s largest wetland areas [76]. Cambodia is facing one of the world’s highest deforestation rate [42, 108, 109]. Therefore, interfaces between anthropic areas and forests are rapidly blurring, increasing the risk of the population being exposed to new emergent diseases and vectors. This change in land use shifts the risk of mosquito-borne disease emergence by changing the relationship between mosquitoes and their hosts, both qualitatively and quantitatively [16, 38, 39]. Urbanisation may facilitate the dispersion of anthropophilic mosquito species into previously unfavorable habitats [51, 52]. This modifies vector-host interactions, and potentially leads to more contact with sylvatic reservoirs of zoonotic pathogens [16]. Consequently anthropophilic, and opportunistic mosquito species may act as bridge vectors between sylvatic and urban pathogen transmission cycles. It is therefore essential to know the diversity of mosquitoes and potential vector species, to be prepared to recognize and cope with emergent or re-emergent arthropod-borne diseases.

In the Greater Mekong sub-region, several mosquito checklists have been produced. In Thailand, at least 464 species were recorded [26, 69, 97], 170 from Laos [46] and 191 from Vietnam [10]. In Cambodia, no such work has yet been undertaken. The only comprehensive studies of generic level groups in the Cambodian context are the works of Klein for *Aedes* (*Neomacleaya*) spp. where 15 species were recorded [32], and *Anopheles* spp. where 37 species were found in the country [26, 33]. Following the socio-political unrest and civil war that the country faced, no entomological activity was done for 25 years [5, 6, 93]. In 2000, Socheath et al. [83] published the results of a five-month vector survey in Kratie province where they identified 13 species of *Anopheles* mosquitoes. None of these species were new for the country. In a study by St-Laurent et al. [88], 22 *Anopheles* species were confirmed in Cambodia and four were new for the country. The use of molecular techniques enabled the authors to assess further and to determine species among several species complexes. More recently Boyer et al. [6] recorded 61 mosquito species from a vectors-survey in Kampong Cham and Tboung Khmum provinces, but no new record was reported in this study. Using available databases [25, 29, 100] and according to the existing literature, 241 species were recorded in Cambodia before the present work.

## Materials and methods

### Study area and specimen collection

This checklist is based on the entomological prospections carried by the medical and veterinary entomology unit at the *Institut Pasteur du Cambodge (IPC)* between 2016 and 2020, and from available online databases. We used, namely, the Walter Reed Biosystematics Unit [100], Mosquito Taxonomic Inventory [25] and MIVEGEC (GECOL/Arim) mosquito collection [29] databases. The names, and their abbreviations, follow the taxonomic nomenclature from Knight and Stone [35, 36] and Reinert [73, 75]. Regarding the names and taxonomy used for the Aedini tribe, we followed the classification provided by Wilkerson et al. [107]

From 2016 to 2020, 11 provinces were investigated as part of five different projects: “*Zikalliance*” (Mondul Kiri province), “*Ecomore 2*” (Kampong Cham), “*PREventing EMerging Pathogenic Threats*” (Rattanak Kiri, Mondul Kiri, Koh Kong, Battambang, Siem Reap, Kampong Speu, Preah Viehar), “*Fonds de Solidarité pour les Projets Innovants*” (Pursat, Battambang, Kampong Soam, Kompong Thom, Preah Vihear provinces), and “*Pagoda*” (Phnom Penh).

Adult mosquitoes were collected using two types of traps baited with dry ice: BG-1 Sentinel^TM^ Mosquito Trap, 7.5–12 Volt (BioQuip, Compton, CA, USA), baited with BG-Lure^®^, and the CDC Mini Light Trap with Incandescent Light. Eggs were collected using an ovitrap, and immature stages prospection was carried out every time a suitable site containing either a temporary or permanent water body was identified. Eggs, larvae and pupae were then bred in the insectarium using a mosquito breeder^®^ (BioQuip, Compton, CA, USA).

### Morphological identification

Each adult specimen was pinned, following the recommendations detailed in Rattanarithikul [61], on plastozoate mounting strips using ×0.15 mm minutens. Specimens were identified using available determination keys [12, 49, 61–69] later confirmed by examination of the original description, or redescription when available, of the species. Identifications were only carried out on adult specimens, mostly on females. Only morphological identification was carried out. Voucher specimens are deposited in the collection of the *Institut Pasteur du Cambodge*, Phnom Penh, Cambodia.

## Results

A total of 230,840 specimens were collected, and 214,945 were identified to the species level. In total, 193 species from 16 genera were collected by the medical and veterinary entomology unit between 2016 and 2020. Forty-nine species were new records for the country. Along with historical data gathered in the literature, the list, distribution and bionomics of mosquitoes is presented hereafter.

## Subfamily Anophelinae Grassi, 1900

### Genus *Anopheles* Meigen, 1818 (53 spp.)

The genus *Anopheles* comprises 480 species worldwide divided among eight subgenera, namely *Anopheles* (190 spp.), *Baimaia* (1 sp.), *Cellia* (224 spp.), *Christya* (2 spp.), *Kerteszia* (12 spp.), *Lophopodomyia* (6 spp.), *Nyssorhynchus* (40 species), and *Stethomyia* (5 species) [25]. Overall, 53 species of *Anopheles* are recorded in Cambodia, belonging to two subgenera: *Anopheles* (28 spp.) and *Cellia* (25 spp.). Enhanced molecular methods have facilitated the identification of *Anopheles* within species complexes and groups; however, in the present work, studies were conducted using only morphological identification, and therefore further work might highlight new species, or revoke existing ones.

#### Subgenus *Anopheles* Meigen, 1818 (28 spp.)

##### *Anopheles* (*Anopheles*) *annandalei* Prashad, 1918

Distribution: Cambodia, India, Indonesia, Nepal and Vietnam [15, 19].

##### *Anopheles* (*Anopheles*) *argyropus* (Swellengrebel, 1914)

Member of the Hyrcanus Group [20, 88]. Distribution: Cambodia, China, India, Indonesia, Malaysia, Myanmar, Thailand, Timor and Vietnam [68].

##### *Anopheles* (*Anopheles*) *baezai* Gater, 1933

Distribution: Cambodia, Guam, Indonesia, Malaysia, Philippines, Singapore, Thailand and Vietnam [1, 68]. Species collected in Cambodia only in the mangrove area in Koh Kong province.

##### *Anopheles* (*Anopheles*) *barbirostris* van der Wulp, 1884

Member of the Barbirostris Complex [88, 92]. Distribution: Bangladesh, Cambodia, China, Guam, India [1], Indonesia, Laos, Malaysia, Myanmar, Nepal, Pakistan, Philippines, Sri Lanka, Thailand, Timor and Vietnam [63, 68]. Larvae found in open sunny to light shade habitats with all types of vegetation. Habitats include stream and river margins and pools, flowing and stagnant ditches, lakes, rice fields, temporary and permanent ground pools, seepage springs, animal footprints, canals, marshes, fish and rock pools. Adults are zoophilic [27].

##### *Anopheles* (*Anopheles*) *barbumbrosus* Strickland & Chowdhury, 1927

Member of the Barbirostris Group [92]. Distribution: Bangladesh, Cambodia, China, India, Indonesia, Malaysia, Nepal, Sri Lanka, Taiwan, Thailand, Timor and Vietnam [1, 68]. Vector of malaria [70]

##### *Anopheles* (*Anopheles*) *bengalensis* Puri, 1930

Distribution: Bangladesh, Cambodia, China, India, Indonesia, Japan, Malaysia, Myanmar, Nepal, Philippines, Taiwan, Thailand and Vietnam [63].

##### *Anopheles* (*Anopheles*) *campestris* Reid, 1962

Member of the Barbirostris Complex [89, 92]. Distribution: Cambodia, China, Guam, Indonesia, Malaysia, Thailand and Vietnam [1, 68]. Larvae can be found in deep-water habitats with vegetation and shade. Habitats include the corners of rice fields, stagnant ditches between rows of coconut palms and earth wells. Adults strongly anthropophagous and bite more inside than outside [70]. Vector of malaria and filariasis [70].

##### *Anopheles* (*Anopheles*) *crawfordi* Reid, 1953

Member of the Hyrcanus Group [20, 89]. Distribution: Cambodia, China, India, Indonesia, Malaysia, Thailand and Vietnam [68].

##### *Anopheles* (*Anopheles*) *dissidens* Taai & Harbach, 2015

Member of the Barbirostris Complex [92]. Distribution: Thailand and Cambodia [105].

##### *Anopheles* (*Anopheles*) *donaldi* Reid, 1962. New record for Cambodia

Member of the Barbirostris Group [92]. Distribution: Indonesia, Laos, Malaysia and Thailand [68]. Collected in Kandal and Kampong Thom provinces. Known as a minor vector of malaria in Borneo [27].

##### *Anopheles* (*Anopheles*) *hodgkini* Reid, 1962

Member of the Barbirostris Group [92]. Distribution: Cambodia, Malaysia, Thailand and Vietnam [68].

##### *Anopheles* (*Anopheles*) *hyrcanus* (Pallas, 1771)

Member of the Hyrcanus Group [20, 89]. Distribution: Afghanistan, Albania, Armenia, Azerbaijan, Bulgaria, Cambodia, China, Croatia, France, Georgia, Greece, Hungary, Iran, Iraq, Italy, Japan, Jordan, Kazakhstan, Kyrgyzstan, Lebanon, Libya, Macedonia, Malaysia, Moldova, Mongolia, Montenegro, Romania, Russia, Serbia, Slovakia, Slovenia, Spain, Sri Lanka, Syria, Tajikistan, Turkey, Turkmenistan, Ukraine and Uzbekistan [60]. However, the presence of this species is doubtful in Southeast Asia [27, 70, 94] and the ITS2 and COI sequences reported by St-Laurent et al. [88] for this species in Cambodia (GenBank No. MW647447-50 and MW603578-82) are very distinct from those of *An. hyrcanus* in Europe.

##### *Anopheles* (*Anopheles*) *insulaeflorum* (Swellengrebel & Swellengrebel de Graaf, 1920)

Distribution: Cambodia, China, India, Indonesia, Malaysia, Myanmar, Philippines, Taiwan, Thailand and Vietnam [68].

##### *Anopheles* (*Anopheles*) *interruptus* Puri, 1929

Distribution: Borneo, Cambodia, China, India, Malaysia, Nepal, Sri Lanka, Thailand and Vietnam [68].

##### *Anopheles* (*Anopheles*) *lesteri* Baisas & Hu, 1936

Member of the Hyrcanus Group [20]. Distribution: Borneo, Cambodia, China, Guam, Hong Kong, Japan, Korea, Malaysia, Philippines, Singapore, Thailand and Vietnam [68]. Immature stages can be found in cool, shaded, groundwater habitats that include marshes, ground pools, ponds, rice fields and other impounded water [93]. Important vector of malaria [93].

##### *Anopheles* (*Anopheles*) *letifer* Sandosham, 1944

Distribution: Cambodia, Indonesia, Malaysia, Singapore, Thailand and Vietnam [68]. Larvae can be found in shaded, dark, acidic water with emergent vegetation or numerous leaves inside the water. Habitats include freshwater swamps, jungle pools and large isolated stream pools. Adults bite at night [27, 70]. Primary malaria vector in Malaysia [70].

##### *Anopheles* (*Anopheles*) *nigerrimus* Giles, 1900

Member of the Hyrcanus Group [20, 89]. Distribution: Bangladesh, Borneo, Cambodia, China, Hong Kong, India, Indonesia, Laos, Malaysia, Myanmar, Nepal, Pakistan, Philippines, Sri Lanka, Thailand, Timor and Vietnam [68]. Species present in lowland and valley areas displaying semi-open large bodies of water with vegetation. Habitats include canals, large open marshes, large stream pools and rice fields [27]. Possible vector of malaria and filariasis [27].

##### *Anopheles* (*Anopheles*) *nitidus* Harrison, Scanlon & Reid, 1973

Member of the Hyrcanus Group [20, 89]. Distribution: Cambodia, China, India, Indonesia, Malaysia, Myanmar, Thailand and Vietnam [68].

##### *Anopheles* (*Anopheles*) *peditaeniatus* (Leicester, 1908)

Member of the Hyrcanus Group [20, 89]. Distribution: Afghanistan, Bangladesh, Bhutan, Cambodia, China, India, Indonesia, Iran, Malaysia, Myanmar, Nepal, Pakistan, Philippines, Sri Lanka, Thailand and Vietnam [68].

##### *Anopheles* (*Anopheles*) *pursati* Laveran, 1902

Member of the Hyrcanus Group [20]. Distribution: Cambodia, Malaysia, Thailand and Vietnam [68].

##### *Anopheles* (*Anopheles*) *roperi* Reid, 1950

Distribution: Cambodia, India, Indonesia, Malaysia and Thailand [68].

##### *Anopheles* (*Anopheles*) *saeungae* Taai & Harbach, 2015

Member of the Barbirostris Complex [88, 92]. Distribution: Cambodia, Indonesia and Thailand [89, 92].

##### *Anopheles* (*Anopheles*) *separatus* (Leicester, 1908)

Distribution: Cambodia, Indonesia, Malaysia, Singapore, Thailand and Vietnam [68].

##### *Anopheles* (*Anopheles*) *sinensis* Wiedemann, 1828

Member of the Hyrcanus Group [20]. Distribution: Cambodia, China, Hong Kong, India, Indonesia, Japan, Korea, Macau, Malaysia, Myanmar, Nepal, Russia, Singapore, Taiwan, Thailand, Timor and Vietnam [68]. Larvae can be found in shallow habitats in freshwater usually with emergent vegetation and exposed to direct sunlight. They are characteristic of open agricultural lands. In mountainous areas, they are confined to the valleys. Females are zoophilic but rarely bite humans [27]. Vector of *Brugia malayi* and secondary vector of malaria in China [27].

##### *Anopheles* (*Anopheles*) *sintonoides* Ho, 1938. New record for Cambodia

Distribution: China, Malaysia, Thailand and Vietnam [68]. In Cambodia, collected in three different provinces: Mondul Kiri, Pailin and Kandal provinces. Larvae were collected from clear black water, in artificial containers (plastic gasoline tank of 1 L).

##### *Anopheles* (*Anopheles*) *umbrosus* (Theobald, 1903)

Distribution: Bangladesh, Cambodia, India, Indonesia, Malaysia, Philippines, Singapore and Thailand [68].

##### *Anopheles* (*Anopheles*) *wejchoochotei* Taai & Harbach, 2015

Member of the Barbirostris Complex [88, 92]. Distribution: Cambodia and Thailand [89, 92].

##### *Anopheles* (*Anopheles*) *whartoni* Reid, 1963

Distribution: Cambodia, Malaysia and Thailand [68]. Larvae can be found in dark peaty water in shady areas. Habitats also include shaded swamps and seepage pools. Adults bite humans outside, generally within 2 h following sunset [27]. Probable poor vector of human malaria and *Wuchereria bancrofti* [27].

#### Subgenus *Cellia* Theobald, 1902 (25 spp.)

##### *Anopheles* (*Cellia*) *aconitus* Dönitz, 1902

Member of the Funestus Group [23, 88]. Distribution: Bangladesh, Cambodia, China, India, Indonesia, Laos, Malaysia, Myanmar, Nepal, Philippines, Sri Lanka, Thailand, Timor and Vietnam [68]. Larvae can be found primarily in flooded rice fields, grassy ponds and stream margins, but also in palm swamps, stream pools, freshwater swamps, rock pools, seepage pools, and ditches. In Thailand, found up to 700 m, and in Indonesia (Java) up to 853 m [27]. Primary vector of malaria [27].

##### *Anopheles* (*Cellia*) *annularis* van der Wulp, 1884

Member of the Annularis Complex [88, 101]. Distribution: Afghanistan, Bangladesh, Cambodia, China, India, Indonesia, Malaysia, Myanmar, Nepal, Pakistan, Philippines, Sri Lanka, Taiwan, Thailand, Timor and Vietnam [68]. Larvae can be found in clear, still water with abundant vegetation. Habitats include ponds, swamps and rice fields. Adults are zoophilic and considered secondary vectors of malaria [70].

##### *Anopheles* (*Cellia*) *baimaii* Sallum, Peyton & Wilkerson, 2005. New record for Cambodia

Member of the Dirus Complex [93]. Distribution: Bangladesh, China, India, Myanmar and Thailand [93]. Collected in Rattanak Kiri province. Primary vector of malaria [91].

###### *Anopheles* (*Cellia*) *culicifacies* Giles, 1901

Member of the Culicifacies Complex [93]. Distribution: Afghanistan, Bahrain, Bangladesh, Cambodia, China, Eritrea, Ethiopia, India, Iran, Iraq, Laos, Myanmar, Nepal, Oman, Pakistan, Sri Lanka, Thailand, Vietnam and Yemen [1, 68]. Immature stages can be found in freshwater irrigation ditches, rain pools, pools in riverbeds, freshly dug pits or holes and wells. Females avoid oviposition sites with emergent vegetation. Can be present at altitudes above 2000 m in Pakistan [27]. Primary vector of malaria [27].

##### *Anopheles* (*Cellia*) *dirus* Peyton & Harrison, 1979

Member of the Dirus Complex [93]. Distribution: Cambodia, China, Laos, Thailand and Vietnam [68]. Immature stages abundant during the rainy season and can be found in several small, shady, temporary ground pools, animal footprints, pools in dry stream beds, springs, streams, ground pools, rock pools, bamboo stumps, and depressions in hollow logs [78]. Primary vector of malaria in forested and hilly-forested areas throughout its distribution range [78]. In Cambodia, this species was only collected in primary forests, in Rattanak Kiri and Preah Vihear provinces.

##### *Anopheles* (*Cellia*) *epiroticus* Linton & Harbach, 2005. New record for Cambodia

Member of the Sundaicus Complex [2]. Distribution: Thailand, Cambodia, Myanmar, Vietnam and Indonesia [91]. In Cambodia, this species was collected in Pursat province. Secondary vector of malaria [91].

##### *Anopheles* (*Cellia*) *indefinitus* Ludlow, 1904

Distribution: Cambodia, China, Guam, Indonesia, Laos, Malaysia, Mariana Islands, Nepal, Philippines, Sri Lanka, Taiwan, Thailand and Vietnam [68]. Primary vector of malaria [88].

##### *Anopheles* (*Cellia*) *jamesii* Theobald, 1901

Distribution: Bangladesh, Cambodia, China, India, Laos, Malaysia, Myanmar, Nepal, Sri Lanka, Thailand and Vietnam [68].

##### *Anopheles* (*Cellia*) *jeyporiensis* James, 1902

Member of the Funestus Group [11, 88]. Distribution: Afghanistan, Bangladesh, Cambodia, China, India, Laos, Macau, Myanmar, Nepal, Taiwan, Thailand and Vietnam [68]. Immature stages can be found in ground habitats with clear, cool, freshwater, with abundant emergent vegetation, and water temperatures within 23–33 °C. Species typical of mountainous areas, which can be found at elevations up to 1829 m [27]. Primary malaria vector [91]. Found naturally infected with *B. malayi* and *W. bancrofti* [27].

##### *Anopheles* (*Cellia*) *karwari* (James, 1903)

Distribution: Bangladesh, Cambodia, China, India, Indonesia, Laos, Malaysia, Myanmar, Nepal, Papua New Guinea, Philippines, Sri Lanka, Thailand and Vietnam [68]. Immature stages can be found in seepages and small streams in open and under light shade in hilly areas. Adults are zoophilic and can be secondary malaria vectors [70].

##### *Anopheles* (*Cellia*) *kochi* Dönitz, 1901

Member of the Kochi Complex [88]. Distribution: Bangladesh, Cambodia, China, India, Indonesia, Laos, Malaysia, Myanmar, Nepal, Philippines, Singapore, Thailand and Vietnam [68]. Primary vector of Malaria [88].

##### *Anopheles* (*Cellia*) *maculatus* Theobald, 1901

Member of the Maculatus Group [2]. Distribution: Bangladesh, Cambodia, China, India, Indonesia, Laos, Malaysia, Myanmar, Nepal, Pakistan, Philippines, Singapore, Sri Lanka, Taiwan, Thailand, Timor and Vietnam [68]. Immature stages can be found in hilly areas in seepage springs and small streams with some sunlight. Found frequently in recently cleared areas with disturbed soil. Adults primarily zoophilic [70]. Primary malaria vector and vector of *W. bancrofti* [70].

##### *Anopheles* (*Cellia*) *minimus* Theobald, 1901

Member of the Minimus Complex [11, 22, 88]. Distribution: Bangladesh, Cambodia, China, India, Indonesia, Japan, Laos, Malaysia, Myanmar, Nepal, Pakistan, Philippines, Sri Lanka, Taiwan, Thailand and Vietnam [1, 68]. Immature stages can be found in small- to moderate-sized streams of clear, cool unpolluted water with partial shade and grassy margins. Other larval habitats include rock pools, sand pools next to streams, seepage pools and springs, stream pools and fallow rice fields with seepage. Females are anthropophilic, endophageous, and are primary malaria vectors [27].

##### *Anopheles* (*Cellia*) *nivipes* (Theobald, 1903)

Member of the Annularis Group [88]. Distribution: Bangladesh, Cambodia, China, India, Laos, Malaysia, Nepal, Thailand and Vietnam [25, 88]. Secondary malaria vector in Cambodia [1, 90]

##### *Anopheles* (*Cellia*) *notanandai* Rattanarithikul & Green, 1987. New record for Cambodia

Member of the Maculatus Group. Distribution: Laos and Thailand [80]. Collected in Kampong Thom province.

##### *Anopheles* (*Cellia*) *pallidus* Theobald, 1901

Member of the Annularis Complex [88, 101]. Distribution: Bangladesh, Cambodia, India, Indonesia, Myanmar, Nepal, Pakistan and Sri Lanka [40, 50, 70]. As noted by Reid [68] and Marcombe et al. [40] some variant forms of *An.* nivipes are similar to *An. pallidus.* Although this species was reported to be identified in Cambodia by St Laurent et al. [88], no DNA sequences of the Cambodian specimens were available in GenBank; therefore the identity of this species will have to be confirmed in the future.

##### *Anopheles* (*Cellia*) *pampanai* Büttiker & Beales, 1959

Member of the Funestus Group [11, 22, 88]. Distribution: Cambodia, Laos, Thailand and Vietnam [68].

##### *Anopheles* (*Cellia*) *philippinensis* Ludlow, 1902

Member of the Annularis Complex [88, 101]. Distribution: Bangladesh, Cambodia, China, India, Indonesia, Laos, Malaysia, Myanmar, Nepal, Philippines, Thailand and Vietnam [68]. Larvae found in clean still, or slowly moving, water with vegetation. Habitats include grassy edges of rice fields, ponds, swamps and irrigation channels. Adults are zoophilic and can be secondary malaria vectors [1, 70, 90]

##### *Anopheles* (*Cellia*) *pseudojamesi* Strickland & Chowdhury, 1927

Distribution: Bangladesh, Cambodia, China, India, Indonesia, Myanmar, Nepal, Thailand and Vietnam [68].

##### *Anopheles* (*Cellia*) *rampae* Harbach & Somboon, 2011

Member of the Maculatus Group [2, 88]. Distribution: Cambodia, India, Thailand and Vietnam [25, 84]. Immature stages can be found in small rock and sand pools exposed to sunlight, often with green algae, along the Mekong River and in hilly forested areas, about 100–400 m above sea-level. Females start biting shortly after sunset (18:00–20:00), are primarily zoophilic, but can sometimes bite humans.

##### *Anopheles* (*Cellia*) *sawadwongporni* Rattanarithikul & Green, 1987

Member of the Maculatus Complex [88]. Distribution: Cambodia, China, India, Laos, Thailand and Vietnam [25, 88].

##### *Anopheles* (*Cellia*) *splendidus* Koidzumi, 1920

Member of the Jamesii Group [88]. Distribution: Afghanistan, Bangladesh, Cambodia, China, India, Laos, Myanmar, Nepal, Pakistan, Taiwan, Thailand and Vietnam [1, 68].

##### *Anopheles* (*Cellia*) *subpictus* Grassi, 1899

Member of the Subpictus Complex [105]. Distribution: Afghanistan, Australia, Bangladesh, Cambodia, China, India, Indonesia, Iran, Malaysia, Maldives, Mariana Islands, Myanmar, Nepal, Papua New Guinea, Pakistan, Philippines, Saudi Arabia, Sri Lanka, Taiwan, Thailand, Timor and Vietnam [68].

##### *Anopheles* (*Cellia*) *tessellatus* Theobald, 1901

Distribution: Bangladesh, Cambodia, China, Guam, India, Indonesia, Laos, Malaysia, Maldives, Myanmar, Nepal, Philippines, Solomon Islands, Sri Lanka, Taiwan, Thailand, Timor and Vietnam [4, 68]. Immature stages are widely distributed but rarely abundant. Larval habitats are usually collections of dirty stagnant water. Adults are primarily zoophilic but can feed on humans [70].

##### *Anopheles* (*Cellia*) *vagus* Dönitz, 1902

Distribution: Afghanistan, Bangladesh, Bhutan, Cambodia, China, Guam, India, Indonesia, Laos, Malaysia, Mariana Islands, Myanmar, Nepal, Philippines, Singapore, Sri Lanka, Thailand and Vietnam [68]. Larvae found in open muddy pools, hoof prints and ditches containing foul, often brackish, water. Adults are strongly zoophagic [70] and are primary vectors of malaria [89].

## Subfamily Culicinae Meigen, 1818

### Tribe: *Aedeomyiini* Theobald, 1901

#### Genus *Aedeomyia* Theobald, 1901 (1 sp.)

The genus *Aedeomyia* includes seven species subdivided into two subgenera: *Aedeomyia* and *Lepiothauma* [25]. Only one species belonging to the subgenus *Aedeomyia* is currently known from Cambodia. These small-sized nocturnal mosquitoes feed mainly on birds and occasionally on humans. Immature stages are often found in association with aquatic vegetation, especially *Pistia*, *Salvinia*, *Eichhornia* and *Potamogeton* [25].

##### Subgenus *Aedeomyia* Theobald, 1901 (1 sp.)

###### *Aedeomyia* (*Aedeomyia*) *catasticta* Knab, 1909

Distribution: Australia, Bangladesh, Cambodia, Fiji, Guam, India, Indonesia, Malaysia, Mariana Islands, Micronesia, Nepal, Papua New Guinea, Palau, Philippines, Solomon Islands, Thailand, Timor and Vietnam [64].

### Tribe: *Aedini* Neveu-Lemaire, 1902

#### Genus *Aedes* Meigen, 1818 (55 spp.)

The genus *Aedes* comprises 79 subgenera [25, 86]. *Aedes* mosquitoes have a worldwide distribution, and some species are considered to be of medical importance as they transmit pathogens. The taxonomic nomenclature follows Wilkerson et al. [107].

##### Subgenus *Aedimorphus* Theobald, 1903 (8 spp.)

###### *Aedes* (*Aedimorphus*) *alboscutellatus* (Theobald, 1905)

Distribution: Australia, Cambodia, India, Indonesia, Japan, Koreas, Laos, Malaysia, Myanmar, Papua New Guinea, Philippines, Solomon Islands, Sri Lanka, Thailand, Timor and Vietnam [64].

###### *Aedes* (*Aedimorphus*) *caecus* (Theobald, 1901)

Distribution: Bangladesh, Cambodia, China, India, Indonesia, Malaysia, Mariana Islands, Myanmar, Nepal, Pakistan, Philippines, Singapore, Thailand and Vietnam [64].

##### *Aedes* (*Aedimorphus*) *culicinus* (Edwards, 1922)

Distribution: Cambodia, India, Indonesia, Pakistan, Thailand and Vietnam [64].

##### *Aedes* (*Aedimorphus*) *mediolineatus* (Theobald, 1901)

Distribution: Cambodia, China, Indonesia, Malaysia, Myanmar, Thailand and Vietnam [64].

##### *Aedes* (*Aedimorphus*) *pampangensis* (Ludlow, 1905). New record for Cambodia

Distribution: India, Indonesia, Nepal, Philippines, Thailand and Vietnam [64]. Species collected in Preah Vihear province.

##### *Aedes* (*Aedimorphus*) *pipersalatus* (Giles, 1902)

Distribution: Bangladesh, Cambodia, India, Laos, Myanmar, Nepal, Pakistan, Sri Lanka and Thailand [64].

##### *Aedes* (*Aedimorphus*) *taeniorhynchoides* (Christophers, 1911)

Distribution: Cambodia, India, Nepal, Pakistan, Sri Lanka, Thailand and Vietnam [71].

##### *Aedes* (*Aedimorphus*) *vexans* (Meigen, 1830)

Distribution: Afghanistan, Algeria, Australia, Austria, Azerbaijan, Bangladesh, Belgium, Belize, Bulgaria, Cambodia, Canada, China, Croatia, Czech Republic, Denmark, Estonia, Fiji, Finland, France, Georgia, Germany, Greece, Guam, Guatemala, Honduras, Hungary, India, Indonesia, Iran, Iraq, Italy, Japan, Kiribati, South Korea, Laos, Latvia, Liberia, Libya, Lithuania, Macedonia, Malaysia, Mauritania, Mexico, Micronesia, Moldova, Mongolia, Montenegro, Morocco, Myanmar, Nepal, Netherlands, New Caledonia, Norway, Pakistan, Papua New Guinea, Philippines, Poland, Portugal, Romania, Russia, Samoa, Saudi Arabia, Serbia, Slovakia, Slovenia, Solomon Islands, South Africa, Spain, Sri Lanka, Sweden, Switzerland, Taiwan, Tajikistan, Thailand, Tonga, Turkey, Tuvalu, Ukraine, United Kingdom, USA, Hawaii, Vanuatu, Vietnam and Yemen [71]. Immature stages can be found in freshwater flood pools, but have also been collected in ditches, swamps, rice fields, and elephant footprints. Habitats usually have little aquatic vegetation or algae. Females bite at night and readily feed on humans and cattle [71]. Species considered to be of medical importance. Confirmed vector of Japanese Encephalitis virus [6] and capable of transmitting Rift valley fever, eastern equine encephalitis virus (EEE), western equine encephalitis virus (WEE), St. Louis encephalitis virus (SLE) and West Nile Virus (WNV) [21, 64, 98]. It is also a vector of dog heartworm [71].

##### Subgenus *Bothaella* Reinert, 1973 (3 spp.)

###### *Aedes* (*Bothaella*) *eldridgei* Reinert, 1973. New record for Cambodia

Distribution: Thailand [64, 74]. Collected in Cambodia in Mondul Kiri province, reared from larvae collected in primary forest, from clear-dark water collected in tree holes.

###### *Aedes* (*Bothaella*) *helenae* Reinert, 1973. New record for Cambodia

Distribution: China and Thailand [64, 74]. Collected in Rattanak Kiri province, using a light trap in the primary forest.

###### *Aedes* (*Bothaella*) *kleini* (Reinert, 1973)

Distribution: known only from Cambodia [71].

##### Subgenus *Bruceharrisonius* Reinert, 2003 (1 sp.)

###### *Aedes* (*Bruceharrisonius*) *aureostriatus* (Doleschall, 1857)

Distribution: Cambodia, Indonesia, Japan, Papua New Guinea, Thailand and Timor [74, 103].

##### Subgenus *Christophersiomyia* Barraud, 1923 (3 spp.)

###### *Aedes* (*Christophersiomyia*) *annulirostris* (Theobald, 1905). New record for Cambodia

Distribution: India, Nepal, Sri Lanka and Thailand [64]. Collected in Mondul Kiri and Rattanak Kiri provinces from larvae reared from dark brown water collected in a large tree hole.

###### *Aedes* (*Christophersiomyia*) *ibis* Barraud, 1931

Distribution: Cambodia, China, India, Malaysia, Philippines, Thailand and Vietnam [64]. Collected in Cambodia in Mondul Kiri and Rattanak Kiri provinces from larvae reared from dark brown water collected in a large tree hole.

###### *Aedes* (*Christophersiomyia*) *thomsoni* (Theobald, 1905). New record for Cambodia

Distribution: Bangladesh, India, Nepal, Pakistan, Philippines, Sri Lanka and Thailand [64, 74]. Collected in Mondul Kiri and Rattanak Kiri provinces from larvae reared from dark brown water collected in a large tree hole.

##### Subgenus *Collessius* Reinert, Harbach & Kitching, 2006 (3 spp.)

###### *Aedes* (*Collessius*) *elsiae* (Barraud, 1923)

Distribution: Cambodia, China, India, Malaysia, Nepal, Taiwan, Thailand and Vietnam [64, 74].

###### *Aedes* (*Collessius*) *macfarlanei* (Edwards, 1914)

Distribution: Cambodia, China, Indonesia, Laos, Malaysia, Thailand and Vietnam [64, 74].

###### *Aedes* (*Collessius*) *pseudotaeniatus* (Giles, 1901)

Distribution: Bangladesh, Cambodia, India, Myanmar, Nepal, Pakistan, Sri Lanka and Thailand [64, 74].

##### Subgenus *Danielsia* (Leicester, 1904) (1 sp.)

###### *Aedes* (*Danielsia*) *albotaeniatus* (Leicester, 1904). New record for Cambodia

Distribution: India, Indonesia, Malaysia, Nepal, Sri Lanka and Thailand [64, 74]. Species collected in Pursat and Kampong Thom Provinces.

##### Subgenus *Downsiomyia* Vargas, 1950 (3 spp.)

###### *Aedes* (*Downsiomyia*) *albolateralis* (Theobald, 1908)

Distribution: Bangladesh, Cambodia, China, India, Indonesia, Japan, Korea, Malaysia, Myanmar, Nepal, Pakistan, Papua New Guinea, Philippines, Sri Lanka, Taiwan, Thailand and Vietnam [64, 74].

###### *Aedes* (*Downsiomyia*) *mikrokopion* Knight & Harrison, 1988. New record for Cambodia

Distribution: Malaysia and Thailand [64, 74]. Collected in Mondul Kiri province.

###### *Aedes* (*Downsiomyia*) *niveoides* (Barraud, 1934)

Distribution: Cambodia, India, Indonesia, Malaysia, Nepal, Thailand and Vietnam [64, 74].

##### Subgenus *Edwardsaedes* Belkin, 1962 (1 sp.)

##### *Aedes* (*Edwardsaedes*) *imprimens* (Walkar, 1860)

Distribution: Australia, Bangladesh, Cambodia, India, Indonesia, Japan, Malaysia, Papua New Guinea, Philippines, Solomon Islands, Thailand and Vietnam [64, 74].

##### Subgenus *Fredwardsius* Reinert, 2000 (1 sp.)

###### *Aedes* (*Fredwardsius*) *vittatus* (Bigot, 1861)

Distribution: Algeria, Angola, Bangladesh, Benin, Botswana, Burkina Faso, Cambodia, Cameroon, Central African Republic, China, Comoros, Cote d’Ivoire, Democratic Republic of the Congo, Djibouti, Ethiopia, France, Gabon, Gambia, Ghana, Guinea, India, Iran, Italy, Kenya, Laos, Liberia, Madagascar, Malawi, Malaysia, Mali, Mozambique, Namibia, Nepal, Niger, Nigeria, Pakistan, Portugal, Saudi Arabia, Senegal, Sierra Leone, Somalia, South Africa, Spain, Sri Lanka, Sudan, South Sudan, Tanzania, Thailand, Tunisia, Uganda, Vietnam, Yemen, Zambia and Zimbabwe [9, 64].

##### Subgenus *Himalaius* Reinert, Harbach & Kitching, 2006 (1 sp.)

###### *Aedes* (*Himalaius*) *gilli* (Barraud, 1924). New record for Cambodia

Distribution: India, Nepal and Thailand [64, 74]. Collected in Mondul Kiri province.

##### Subgenus *Hulecoeteomyia* Theobald, 1904 (3 spp.)

###### *Aedes* (*Hulecoeteomyia*) *chrysolineatus* (Theobald, 1907)

Distribution: Bangladesh, Cambodia, India, Indonesia, Laos, Malaysia, Nepal, Philippines, Sri Lanka, Thailand and Vietnam [64].

###### *Aedes* (*Hulecoeteomyia*) *harveyi* (Barraud, 1923)

Distribution: Cambodia, China, India, Indonesia, Malaysia, Nepal, Sri Lanka, Taiwan, Thailand and Vietnam [64]. Collected in Cambodia only from the primary forest of Mondul Kiri, reared from larvae collected in clear water from an artificial container in a bamboo forest.

###### *Aedes* (*Hulecoeteomyia*) *saxicola* Edwards, 1922

Distribution: Cambodia, China, India, Indonesia, Malaysia, Philippines, Thailand and Vietnam [64].

##### Subgenus *Kenknightia* Reinert, 1990 (1 sp.)

###### *Kenknightia dissimilis* (Leicester, 1908). New record for Cambodia

Distribution: Bangladesh, China, India, Malaysia, Nepal, Thailand and Vietnam [64, 74]. Collected in Mondul Kiri province.

##### Subgenus *Lorrainea* Belkin, 1962 (2 spp.)

###### *Aedes* (*Lorrainea*) *amesii* (Ludlow, 1903). New record for Cambodia

Distribution: India, Indonesia, Malaysia, Philippines, Singapore and Thailand [64]. Collected in the mangrove forest in Koh Kong province.

###### *Aedes* (*Lorrainea*) *fumidus* Edwards, 1928

Distribution: Cambodia, India, Indonesia, Malaysia, Philippines, Singapore and Thailand [64].

##### Subgenus *Mucidus* Theobald, 1901 (2 spp.)

###### *Aedes* (*Mucidus*) *laniger* (Wiedemann, 1820)

Distribution: Cambodia, India, Indonesia, Malaysia, Philippines, Singapore, Thailand and Vietnam [64].

###### *Aedes* (*Mucidus*) *quasiferinus* Mattingly, 1961

Distribution: Cambodia, India, Indonesia, Malaysia, Singapore, Sri Lanka and Thailand [64].

##### Subgenus *Neomelaniconion* Newstead, 1907 (1 sp.)

###### *Aedes* (*Neomelaniconion*) *lineatopennis* (Ludlow, 1905)

Distribution: Australia, Bangladesh, Benin, Borneo, Burkina Faso, Cambodia, China, Congo, Gabon, Ghana, India, Indonesia, Kenya, South Korea, Laos, Malaysia, Namibia, Nepal, Nigeria, Nigeria, Pakistan, Philippines, Russia, South Africa, Sri Lanka, Thailand, Timor, Togo and Vietnam [64].

##### Subgenus *Ochlerotatus* (Skuse, 1889) (1 sp.)

###### *Aedes* (*Ochlerotatus*) *vigilax* (Skuse, 1889). New record for Cambodia

Distribution: Australia, Fiji, Indonesia, Japan, Malaysia, New Caledonia, Papua New Guinea, Philippines, Seychelles, Solomon Islands, Taiwan, Thailand, Timor, Tonga, Vanuatu and Vietnam [64]. Collected in Kampong Cham province.

##### Subgenus *Paraedes* Edwards, 1934 (2 spp.)

###### *Aedes* (*Paraedes*) *ostentatio* (Leicester, 1908)

Distribution: Cambodia, India, Indonesia, Laos, Malaysia, Philippines, Sri Lanka, Thailand and Vietnam [64].

###### *Aedes* (*Paraedes*) *thailandensis* Reinert, 1976. New record for Cambodia

Distribution: Thailand and Vietnam [64]. Collected in Rattanak Kiri and Pursat provinces.

##### Subgenus *Petermattinglyius* Reinert, Harbach & Kitching, 2009 (1 sp.)

###### *Aedes* (*Petermattinglyius*) *scanloni* Reinert, 1970

Distribution: Cambodia and Thailand [64].

##### Subgenus *Phagomyia* Theobald, 1905 (4 spp.)

###### *Aedes* (*Phagomyia*) *assamensis* (Theobald, 1908)

Distribution: Bangladesh, Cambodia, China, India, Indonesia, Japan, Nepal, Thailand and Vietnam [64].

###### *Aedes* (*Phagomyia*) *feegradei* (Barraud, 1934). New record for Cambodia

Distribution: Japan, Myanmar, Nepal and Thailand [64]. Collected in Mondul Kiri province.

###### *Aedes* (*Phagomyia*) *khazani* Edwards, 1922

Distribution: Bangladesh, Cambodia, India, Malaysia, Nepal, Thailand and Vietnam [64].

###### *Aedes* (*Phagomyia*) *prominens* (Barraud, 1923)

Distribution: Cambodia, China, India, Indonesia, Malaysia, Nepal, Thailand and Vietnam [64]. Collected in primary forests from Mondul Kiri province and from the mangrove forest of Koh Kong province. Larvae were reared from clear water collected in tree hole.

##### Subgenus *Scutomyia* Theobald, 1904 (1 sp.)

###### *Aedes* (*Scutomyia*) *albolineatus* (Theobald, 1904)

Distribution: Cambodia, China, India, Indonesia, Malaysia, Mariana Islands, Papua New Guinea, Philippines, Singapore, Solomon Islands, Taiwan, Thailand and Vietnam [64]. Collected in Cambodia in Mondul Kiri province, from larvae found in plastic gasoline container, from clear yellowish water.

##### Subgenus *Stegomyia* Theobald, 1901 (10 spp.)

###### *Aedes* (*Stegomyia*) *aegypti* (Linnaeus, 1762)

Distribution: Afghanistan, Algeria, Angola, Anguilla, Antigua and Barbuda, Argentina, Australia, Bahamas, Bangladesh, Barbados, Belize, Benin, Bermuda, Bhutan, Bolivia, Borneo, Bosnia and Herzegovina, Botswana, Brazil, Burkina Faso, Cambodia, Cameroon, Cape Verde, Cayman Islands, Central African Republic, Chad, Chile, China, Colombia, Comoros, Congo, Costa Rica, Cote d’Ivoire, Croatia, Cuba, Democratic Republic of the Congo, Djibouti, Dominica, Dominican Republic, Ecuador, Egypt, El Salvador, Equatorial Guinea, Eritrea, Ethiopia, Fiji, France, French Guiana, French Polynesia, Gabon, Gambia, Georgia, Ghana, Grenada, Guadeloupe, Guam, Guatemala, Guinea, Guinea Bissau, Guyana, Haiti, Honduras, India, Indonesia, Iran, Iraq, Israel, Italy, Jamaica, Japan, Kenya, Kiribati, Laos, Lebanon, Liberia, Libya, Madagascar, Malawi, Malaysia, Maldives, Mali, Mariana Islands, Marshall Islands, Martinique, Mauritania, Mauritius, Mexico, Micronesia, Montserrat, Morocco, Mozambique, Myanmar, Namibia, Nauru, Nepal, Netherlands, New Caledonia, Nicaragua, Niger, Nigeria, Niue, Oman, Pakistan, Papua New Guinea, Panama, Paraguay, Peru, Philippines, Portugal, Puerto Rico, Reunion, Rwanda, Saint Kitts and Nevis, Saint Lucia, Saint Vincent and the Grenadines, Samoa, São Tomé-and-Príncipe, Saudi Arabia, Senegal, Seychelles, Sierra Leone, Singapore, Solomon Islands, Somalia, South Africa, Spain, Sri Lanka, Sudan, South Sudan, Suriname, Syria, Taiwan, Tanzania, Thailand, Timor, Togo, Tonga, Trinidad and Tobago, Tunisia, Turkey, Tuvalu, Uganda, Uganda, United Kingdom, USA, Hawaii, Uruguay, Vanuatu, Venezuela, Vietnam, Virgin Islands, Yemen, Zambia and Zimbabwe [25, 64]. *Aedes aegypti* is an anthropic species using any type of artificial container. Away from urban areas, the species tends to favor pools in river beds, tree stumps, tree holes and natural containers. Females are primarily day biters and readily enter buildings to feed. Considered of medical importance, it is known worldwide as a vector of YFV, DENV, ZIKV, CHIKV and at least 16 other arboviruses [13, 95].

###### *Aedes* (*Stegomyia*) *albopictus* (Skuse, 1895)

Distribution: Albania, Algeria, Argentina, Australia, Austria, Bangladesh, Barbados, Belgium, Belize, Bermuda, Bhutan, Bolivia, Borneo, Bosnia and Herzegovina, Brazil, British Indian Ocean Territory, Bulgaria, Cambodia, Cameroon, Cayman Islands, Central African Republic, China, Cocos Islands, Colombia, Comoros, Congo, Costa Rica, Cote d’Ivoire, Croatia, Cuba, Czech Republic, Democratic Republic of the Congo, Dominican Republic, El Salvador, Equatorial Guinea, Fiji, France, French Polynesia, Gabon, Georgia, Germany, Greece, Guam, Guatemala, Haiti, Honduras, Hong Kong, India, Indonesia, Israel, Italy, Japan, South Korea, Laos, Lebanon, Macau, Madagascar, Malaysia, Maldives, Malta, Mariana Islands, Marshall Islands, Mauritius, Mexico, Montenegro, Myanmar, Nepal, Netherlands, New Caledonia, New Zealand, Nicaragua, Nigeria, Pakistan, Palau, Panama, Papua New Guinea, Paraguay, Philippines, Polynesian Islands, Wallis & Futuna, Puerto Rico, Reunion, Romania, Russia, Samoa, San Marino, Seychelles, Singapore, Slovakia, Slovenia, Solomon Islands, South Africa, Spain, Sri Lanka, Switzerland, Syria, Taiwan, Thailand, Timor, Tonga, Trinidad and Tobago, Turkey, Turkey, Tuvalu, USA, Hawaii, Uruguay, Vanuatu, Venezuela and Vietnam [64]. Immature stages are found in natural containers, including tree holes, bamboo stumps, coconut shells, rockholes, palm fronds, and leaf axils. They can also be found in all varieties of artificial containers and will breed indoors. Known vector of at least Dengue (serotypes 1, 2, 3, 4), Chikungunya, Zika, Arumowot, Bujaru, Bussuquara, Cache Valley, Chandipura, Chilibre, Eastern Equine encephalitis, Getah, Icoaraci, Ilheus, Itaporanga, Jamestown Canyon, Japanese Encephalitis, Karimabad, Keystone, Kokobera, La Crosse, Mayaro, Nodamura, Oropuche, Orungo, Pacui, Potosi, Rift Valley fever, Ross River, Salehabad, San Angelo, St. Louis encephalitis, Tensaw, Trivittatus, Uganda S., Urucuri, Usutu, Venezuelan equine encephalitis, Western equine encephalitis, West Nile, and Yellow fever [6, 28, 51, 53].

###### *Aedes* (*Stegomyia*) *annandalei* (Theobald, 1910)

Distribution: Bangladesh, Cambodia, China, India, Indonesia, Myanmar, Nepal, Papua New Guinea, Taiwan, Thailand and Vietnam [64].

###### *Aedes* (*Stegomyia*) *desmotes* (Giles, 1904)

Distribution: Cambodia, India, Indonesia, Malaysia, Philippines, Singapore, Taiwan, Thailand and Vietnam [64].

###### *Aedes* (*Stegomyia*) *gardnerii imitator* (Leicester, 1908)

Distribution: Cambodia, Indonesia, Malaysia, Philippines, Singapore and Vietnam [64].

###### *Aedes* (*Stegomyia*) *malayensis* Colless, 1962

Distribution: Cambodia, India, Malaysia, Singapore, Taiwan, Thailand and Vietnam [64]. Species of medical importance, vector of dengue.

###### *Aedes* (*Stegomyia*) *malikuli* Huang, 1973. New record for Cambodia

Distribution: Taiwan and Thailand [64]. Collected in Mondul Kiri province only from larvae collected in transparent – but colored with tannin – water in dead bamboo trees.

###### *Aedes (Stegomyia) pseudalbopictus* (Borel, 1928). New record for Cambodia

Distribution: India, Indonesia, Laos, Malaysia, Myanmar, Nepal, Taiwan, Thailand and Vietnam [64]. Collected in Mondul Kiri province only, from larvae collected in transparent – but colored with tannin – water in dead bamboo trees and from large tree holes.

###### *Aedes* (*Stegomyia*) *scutellaris* (Walker, 1859)

Distribution: Australia, Cambodia, Fiji, Guam, India, Indonesia, Mariana Islands, Micronesia, Papua New Guinea, Philippines, Sri Lanka, Tuvalu and Vanuatu [64]. Immature stages have been collected from coconut shells and artificial containers [28]. Possible vector of dengue [28].

###### *Aedes* (*Stegomyia*) *w-albus* (Theobald, 1905). New record for Cambodia

Distribution: Bangladesh, China, India, Indonesia, Malaysia, Nepal, Pakistan, Sri Lanka, Thailand and Vietnam [64]. Collected in Mondul Kiri province.

##### Subgenus *Tanakaius* Reinert, Harbach & Kitching, 2004 (1 sp.)

###### *Aedes* (*Tanakaius*) *togoi* (Theobald, 1907)

Distribution: Cambodia, Canada, China, Japan, Koreas, Malaysia, Russia, Taiwan, Thailand, USA and Vietnam [64]. Larvae are common in coastal regions. Larvae usually occur in tidal pools or rock pools of salt and brackish waters, also occasionally found in containers containing freshwater. Females readily bite humans through the day [94]. Possible vector of *B. malayi, W. bancrofti, Dirofilaria immitis* [94].

##### Subgenus *Rhinoskusea* Edwards, 1929 (1 sp.)

###### *Aedes* (*Rhinoskusea*) *longirostris* (Leicester, 1908). New record for Cambodia

Distribution: Australia, India, Indonesia, Malaysia, Papua New Guinea, Philippines, Singapore, Thailand and Vietnam [64]. Collected in the mangrove forest of Koh Kong province.

#### Genus *Armigeres* Theobald, 1901 (26 spp.)

The genus *Armigeres* is composed of 58 species divided among two subgenera: *Armigeres* and *Leicesteria* [25]. Both are represented in Cambodia by 12 and 14 species, respectively. Immature stages are found in small collections of water, particularly those containing foul water or with a high organic content. They occur in hollow logs, rock holes, tree holes, stump holes, bamboo, Pandanus axils, sago palm and banana stumps, fruit shells and husks, fallen leaves and spathes, flower bracts, pitcher plants, artificial containers that contain organic matter and small collections of groundwater. Larvae are partially carnivorous. Adults occur primarily in forested and plantation areas and are mainly diurnal and active at dusk. Females of many species bite humans.

##### Subgenus *Armigeres* Theobald, 1901 (12 spp.)

###### *Armigeres* (*Armigeres*) *aureolineatus* (Leicester, 1908)

Distribution: Cambodia, China, India, Laos, Malaysia, Nepal, Philippines, Sri Lanka, Thailand and Vietnam [64]. Immature stages can be found in coconut shells.

###### *Armigeres* (*Armigeres*) *confusus* Edwards, 1915. New record for Cambodia

Distribution: Indonesia, Malaysia, Singapore and Thailand [64]. Collected in Mondul Kiri and Pailin provinces.

###### *Armigeres* (*Armigeres*) *durhami* (Edwards, 1917)

Distribution: Cambodia, India, Indonesia, Laos, Malaysia, Nepal, Thailand and Vietnam [64].

###### *Armigeres* (*Armigeres*) *foliatus* Brug, 1931. New record for Cambodia

Distribution: Indonesia, Laos and Thailand [46, 64]. Collected in Mondul Kiri and Preah Vihear provinces.

###### *Armigeres* (*Armigeres*) *kesseli* Ramalingam, 1987. New record for Cambodia

Distribution: India, Malaysia, Nepal and Thailand [64]. Collected in Mondul Kiri and Siem Reap provinces.

###### *Armigeres* (*Armigeres*) *kuchingensis* Edwards, 1915

Distribution: Bangladesh, Cambodia, India, Indonesia, Laos, Malaysia, Nepal, Philippines, Thailand and Vietnam [64].

###### *Armigeres* (*Armigeres*) *laoensis* Toma & Miyagi, 2003. New record for Cambodia

Distribution: Laos [25, 46]. Collected in Mondul Kiri province.

###### *Armigeres* (*Armigeres*) *malayi* (Theobald, 1901). New record for Cambodia

Distribution: China, Indonesia, Papua New Guinea, Philippines, Thailand, Timor and Vietnam [64]. Collected in Pursat and Preah Vihear provinces.

###### *Armigeres* (*Armigeres*) *maximus* Edwards, 1922. New record for Cambodia

Male unknown. Distribution: Indonesia, Malaysia and Thailand [64]. Collected in Pailin province.

###### *Armigeres* (*Armigeres*) *moultoni* Edwards, 1914

Distribution: Cambodia, Indonesia, Laos, Malaysia, Thailand and Vietnam [64].

###### *Armigeres* (*Armigeres*) *subalbatus* (Coquillett, 1898)

Distribution: Bangladesh, Cambodia, China, India, Indonesia, Japan, Korea, Laos, Malaysia, Myanmar, Nepal, Pakistan, Philippines, Sri Lanka, Taiwan, Thailand and Vietnam [64]. Immature stages are found in various container habitats containing nutrient-rich and polluted water. Females bite throughout the day, with peaks at dawn and dusk [94]. Vector of *W. bancrofti* [94]. In Cambodia, the larvae were collected mostly in banana stumps.

###### *Armigeres* (*Armigeres*) *theobaldi* Barraud, 1934. New record for Cambodia

Distribution: India, Laos, Myanmar and Thailand [64]. Collected in Mondul Kiri and Kampong Cham provinces.

##### Subgenus *Leicesteria* Theobald, 1904 (14 spp.)

###### *Armigeres* (*Leicesteria*) *annulipalpis* (Theobald, 1910)

Distribution: Cambodia, China, India, Indonesia, Myanmar and Thailand [64].

###### *Armigeres* (*Leicesteria*) *annulitarsis* (Leicester, 1908)

Distribution: Bangladesh, Cambodia, India, Indonesia, Laos, Malaysia, Nepal, Taiwan, Thailand and Vietnam [64]. Collected in primary forests in Mondul Kiri and Rattanak Kiri provinces using light traps.

###### *Armigeres* (*Leicesteria*) *balteatus* Macdonald, 1960. New record for Cambodia

Distribution: Indonesia, Malaysia, Philippines and Thailand [64]. Collected in Mondul Kiri province, from larvae collected in clear water in dead bamboo holes.

###### *Armigeres* (*Leicesteria*) *cingulatus* (Leicester, 1908)

Distribution: Cambodia, India, Malaysia, Myanmar, Thailand and Vietnam [64].

###### *Armigeres* (*Leicesteria*) *dentatus* Barraud, 1927

Distribution: Bangladesh, Cambodia, India, Malaysia, Nepal, Thailand and Vietnam [64].

###### *Armigeres* (*Leicesteria*) *digitatus* (Edwards, 1914)

Distribution: Bangladesh, Cambodia, India, Indonesia, Malaysia, Nepal, Philippines, Taiwan, Thailand and Vietnam [64].

###### *Armigeres* (*Leicesteria*) *dolichocephalus* (Leicester, 1908)

Distribution: Cambodia, India, Indonesia, Laos, Malaysia, Nepal, Thailand and Vietnam [64]. Species collected only in forests in Mondul Kiri province.

###### *Armigeres* (*Leicesteria*) *flavus* (Leicester, 1908)

Distribution: Bangladesh, Cambodia, China, India, Indonesia, Laos, Malaysia, Myanmar, Nepal, Philippines, Taiwan, Thailand and Vietnam [64].

###### *Armigeres* (*Leicesteria*) *inchoatus* Barraud, 1927. New record for Cambodia

Distribution: Bangladesh, India, Malaysia, Nepal and Thailand [64]. Collected in Mondul Kiri province.

###### *Armigeres* (*Leicesteria*) *longipalpis* (Leicester, 1904)

Distribution: Cambodia, India, Indonesia, Laos, Malaysia, Thailand and Vietnam [64]. Collected in Cambodia in Mondul Kiri province, from larvae collected in transparent water colored with tannin, from dead bamboo tree holes.

###### *Armigeres* (*Leicesteria*) *magnus* (Theobald, 1908)

Distribution: Bangladesh, Cambodia, China, India, Indonesia, Laos, Macau, Malaysia, Myanmar, Nepal, Philippines, Sri Lanka, Taiwan, Thailand and Vietnam [64]. Collected in Cambodia by humans landing catch from the primary forest in Mondul Kiri province.

###### *Armigeres* (*Leicesteria*) *omissus* (Edwards, 1914)

Distribution: Bangladesh, Cambodia, China, India, Indonesia, Malaysia, Nepal, Philippines, Sri Lanka, Taiwan and Thailand [64].

###### *Armigeres* (*Leicesteria*) *pectinatus* (Edwards, 1914)

Distribution: Cambodia, Indonesia, Laos, Malaysia, Philippines, Thailand and Vietnam [64].

###### *Armigeres* (*Leicesteria*) *traubi* Macdonald, 1960. New record for Cambodia

Distribution: Malaysia and Thailand [64]. Collected in Mondul Kiri province.

#### Genus *Heizmannia* Ludlow, 1905 (10 spp.)

This genus comprises 40 species divided among two subgenera (*Heizmannia*, *Mattinglyia*) [25, 47]. Both are present in Cambodia, hosting eight and two species, respectively. Larvae are found mainly in tree holes and bamboos, but some species are occasionally found in small ground pools. Little is known about the biology of the adults: they are apparently active during daytime in forests where females readily bite humans [41]. No species is reported to be of medical importance.

##### Subgenus *Heizmannia* Ludlow, 1905 (8 spp.)

###### *Heizmannia* (*Heizmannia*) *aureochaeta* (Leicester, 1908)

Distribution: Cambodia, India, Malaysia and Thailand [64]. Immature stages can be found in bored bamboos and in flood pools in bamboo groves [41]. Adults are known to bite humans during the daytime [41].

###### *Heizmannia* (*Heizmannia*) *chengi* Lien, 1968. New record for Cambodia

Distribution: India, Malaysia, Taiwan and Thailand [64]. Collected in Mondul Kiri and Siem Reap provinces.

###### *Heizmannia* (*Heizmannia*) *communis* (Leicester, 1908). New record for Cambodia

Distribution: Indonesia, Malaysia, Thailand and Vietnam [64]. Collected in Pursat province.

###### *Heizmannia* (*Heizmannia*) *complex* (Theobald, 1910)

Distribution: Cambodia, India, Laos, Malaysia, Myanmar, Thailand and Vietnam [64]. Immature stages can be found in tree holes and bored bamboo [41]. Adults can feed on humans in dense forests during the daytime.

###### *Heizmannia* (*Heizmannia*) *demeilloni* Mattingly, 1970. New record for Cambodia

Distribution: Myanmar, Nepal and Thailand [64]. Collected in Rattanak Kiri, Kampong Saom, Preah Vihear and Pursat provinces.

###### *Heizmannia* (*Heizmannia*) *mattinglyi* Thurman, 1959

Immature stages and adult male unknown. Distribution: Cambodia and Thailand [64]. Holotype bred from tree hole, and paratype collected from water in a split bamboo [41]. Adults are known to bite humans [41].

###### *Heizmannia* (*Heizmannia*) *reidi* Mattingly, 1957

Distribution: Cambodia, India, Malaysia, Myanmar, Nepal, Taiwan, Thailand and Vietnam [64]. Immature stages can be found in tree holes, bamboo internodes, ground pools and bamboo stumps. Adults readily bite humans [41].

###### *Heizmannia* (*Heizmannia*) *scintillans* Ludlow, 1905

Distribution: Cambodia, Indonesia, Malaysia, Philippines, Singapore, Thailand and Vietnam [64]. Immature stages can be found in tree holes, bamboo internodes and banana axils water [41]. Adults can bite humans in shaded forests.

##### Subgenus *Mattinglyia* Lien, 1968 (2 spp.)

###### *Heizmannia* (*Mattinglyia*) *achaetae* (Leicester, 1908)

Distribution: Cambodia, Indonesia, Malaysia and Thailand [64]. Adults can feed on humans in shaded forests. Immature stages were found in water from tree holes and fallen coconut leaves [41].

###### *Heizmannia* (*Mattinglyia*) *catesi* Lien, 1968

Distribution: Cambodia, Taiwan and Thailand [64]. Adults can feed on humans in shaded forests during the daytime. Only known breeding place for immature stages is bamboo stumps [41].

#### Genus *Verrallina* Theobald, 1903 (19 spp.)

The genus *Verrallina* includes 95 species distributed worldwide, and separated in three subgenera: *Verrallina, Harbachius*, and *Neomacleaya* [25]. These three subgenera are present in Cambodia, hosting 2, 5 and 12 species, respectively. The immature stages are found in temporary ground pools. Several species are reported to bite humans during the daytime, often in shaded places or in the vicinity of forests.

##### Subgenus *Harbachius* Reinert, 1999 (5 spp.)

###### *Verrallina* (*Harbachius*) *abdita* (Barraud, 1931)

Distribution: Cambodia, India and Thailand [64].

###### *Verrallina* (*Harbachius*) *fragilis* Leicester, 1908

Immature stages unknown [17]. Distribution: Cambodia and Malaysia [72].

###### *Verrallina* (*Harbachius*) *indecorabilis* Leicester, 1908

Immature stages unknown [17]. Distribution: Cambodia, Malaysia and Thailand [64].

###### *Verrallina* (*Harbachius*) *stunga* (Klein, 1973)

Distribution: only known from Cambodia [32].

###### *Verrallina* (*Harbachius*) *yusafi* (Barraud, 1931). New record for Cambodia

Male unknown [25]. Distribution: India, Sri-Lanka and Thailand [25, 32]. Species collected in Kandal and Kampong Tom provinces.

##### Subgenus *Neomacleaya* Theobald, 1907 (12 spp.)

###### *Verrallina* (*Neomacleaya*) *adusta* (Laffoon, 1946)

Distribution: Cambodia, Malaysia, Philippines and Thailand [64].

###### *Verrallina* (*Neomacleaya*) *andamanensis* (Edwards, 1922)

Distribution: Andaman Islands, Bangladesh, Cambodia, India, Indonesia, Malaysia, Philippines, Singapore, Thailand and Vietnam [64]. Immature stages can be collected in pools in dry stream beds and shaded road ruts, and in puddles [17].

###### *Verrallina* (*Neomacleaya*) *clavata* (Barraud, 1931)

Immature stages unknown [17]. Distribution: Cambodia, India, Thailand and Vietnam [64]. Adults have been collected in rainforests in baited traps (type of bait unspecified) [17].

###### *Verrallina* (*Neomacleaya*) *cretata* (Delfinado, 1967)

Distribution: Cambodia, Malaysia and Thailand [64]. Larvae can be collected in flood pools in rainforests [17].

###### *Verrallina* (*Neomacleaya*) *cyrtolabis* (Edwards, 1928)

Female unknown [17]. Distribution: Cambodia, Malaysia, Singapore and Thailand [64]. Immature stages can be collected in mangrove areas [17].

###### *Verrallina* (*Neomacleaya*) *komponga* (Klein, 1973)

Distribution: only known from Cambodia [32].

###### *Verrallina* (*Neomacleaya*) *notabilis* (Delfinado, 1967)

Immature stages and adult male unknown [17]. Distribution: Cambodia and Thailand [64].

###### *Verrallina* (*Neomacleaya*) *phnoma* (Klein, 1973)

Distribution: Cambodia and Thailand [32, 64].

###### *Verrallina* (*Neomacleaya*) *rara* (Delfinado, 1968)

Distribution: Cambodia, Indonesia and Malaysia [32].

###### *Verrallina* (*Neomacleaya*) *taeniata* (Leicester, 1908)

Distribution: Cambodia, Indonesia, Malaysia and Thailand [64].

###### *Verrallina* (*Neomacleaya*) *unca* (Theobald, 1901)

Larvae unknown [17]. Distribution: Cambodia, India, Indonesia, Malaysia, Philippines, Thailand, Vietnam [64]. Immature stages can be collected in various types of temporary ground pools and in axils of the *Colocasia* plants. Adults are diurnal and can readily bite humans [17].

###### *Verrallina* (*Neomacleaya*) *vallistris* (Barraud, 1928)

Immature stages unknown [17]. Distribution: Cambodia, India, Myanmar and Thailand [64].

##### Subgenus *Verrallina* Theobald, 1903 (2 spp.)

###### *Verrallina* (*Verrallina*) *butleri* (Theobald, 1901)

Distribution: Australia, Borneo, Cambodia, India, Indonesia, Malaysia, Myanmar, Philippines, Singapore, Sri Lanka, Thailand and Vietnam [64].

###### *Verrallina* (*Verrallina*) *dux* (Dyar & Shannon, 1925)

Distribution: Cambodia, China, India, Laos, Malaysia, Philippines, Thailand and Vietnam [64]. Adults collected in crab holes, while larvae were found in footprints in ricefields near mangrove swamps in salt marshes and in brackish waters [17].

#### Genus *Culex* Linnaeus, 1758 (57 spp.)

The genus *Culex* consists of 779 species divided among 26 subgenera [25]. In Cambodia, five subgenera are represented: *Culex* (15 species), *Culiciomyia* (five species), *Eumelanomyia* (11 species), *Lophoceraomyia* (18 species) and *Oculeomyia* (three species). *Culex* larvae occur primarily in semi- or permanent bodies of groundwater and can be found in artificial containers. *Culex* females feed mainly during the night. Some species are reported to be of medical importance and can be vectors of numerous arboviruses and filariosis [25].

##### Subgenus *Culex* Linnaeus, 1758 (16 spp.)

###### *Culex* (*Culex*) *alienus* Colless, 1957

Distribution: Cambodia, Malaysia, Singapore, Thailand and Vietnam [66].

###### *Culex* (*Culex*) *alis* Theobald, 1903

Distribution: Cambodia, Guam, Indonesia, Malaysia, Papua New Guinea, Philippines, Singapore, Taiwan, Thailand and Vietnam [66].

###### *Culex* (*Culex*) *annulus* Theobald, 1901

Distribution: Cambodia, China, Hong Kong, Indonesia, Malaysia and Philippines [97].

###### *Culex* (*Culex*) *barraudi* Edwards, 1922

Distribution: Cambodia, China, India, Nepal, Pakistan, Sri Lanka and Thailand [66].

###### *Culex* (*Culex*) *fuscocephala* Theobald, 1907

Distribution: Bangladesh, Cambodia, China, Guam, Hong Kong, India, Indonesia, Japan, Laos, Malaysia, Myanmar, Nepal, Pakistan, Philippines, Singapore, Sri Lanka, Thailand, Timor and Vietnam [66]. Species of medical importance, confirmed vector of Japanese encephalitis virus [6].

###### *Culex* (*Culex*) *gelidus* Theobald, 1901

Distribution: Bangladesh, Cambodia, China, Hong Kong, India, Indonesia, Japan, Laos, Malaysia, Myanmar, Nepal, Pakistan, Papua New Guinea, Philippines, Sri Lanka, Taiwan, Thailand and Vietnam [66]. Larvae can be found in a variety of temporary and semi-permanent groundwater habitats such as pools, puddles and small streams, but also occasionally found in artificial containers such as barrels and water tanks. Females prefer to bite large domestic animals [7]. Species of medical importance, confirmed vector for Japanese encephalitis virus [6].

###### *Culex* (*Culex*) *hutchinsoni* Barraud, 1924

Distribution: Bangladesh, Cambodia, India, Laos, Malaysia, Myanmar, Nepal, Pakistan, Philippines, Singapore, Thailand and Vietnam [66].

###### *Culex* (*Culex*) *mimulus* Edwards, 1915

Distribution: Australia, Bangladesh, Cambodia, China, Hong Kong, India, Indonesia, Malaysia, Myanmar, Nepal, Pakistan, Papua New Guinea, Philippines, Singapore, Sri Lanka, Taiwan, Thailand and Vietnam [66].

###### *Culex* (*Culex*) *perplexus* Leicester, 1908

Distribution: Cambodia, India, Indonesia, Malaysia, Papua New Guinea, Philippines, Singapore and Thailand [62].

###### *Culex* (*Culex*) *pseudovishnui* Colless, 1957

Distribution: Bangladesh, Cambodia, China, Hong Kong, India, Indonesia, Iran, Iraq, Japan, Korea, Laos, Macau, Malaysia, Nepal, Pakistan, Papua New Guinea, Philippines, Singapore, Sri Lanka, Taiwan, Thailand and Vietnam [77].

###### *Culex* (*Culex*) *quinquefasciatus* Say, 1823

Distribution: Afghanistan, Angola, Anguilla, Antigua and Barbuda, Argentina, Australia, Bahamas, Bahrain, Bangladesh, Barbados, Belize, Bolivia, Brazil, Burkina Faso, Cambodia, Cameroon, Cayman Islands, Central African Republic, Chile, China, Colombia, Comoros, Congo, Cook Islands, Costa Rica, Cote d’Ivoire, Croatia, Cuba, Democratic Republic of the Congo, Djibouti, Dominica, Dominican Republic, Ecuador, El Salvador, Equatorial Guinea, Ethiopia, Fiji, France, France, French Guiana, French Polynesia, Gabon, Gambia, Greece, Grenada, Guadeloupe, Guam, Guatemala, Guyana, Haiti, Honduras, India, Indonesia, Iran, Iraq, Jamaica, Japan, Kenya, Kiribati, Kuwait, Laos, Liberia, Madagascar, Malaysia, Maldives, Mali, Mariana Islands, Marshall Islands, Martinique, Mauritania, Mauritius, Mexico, Micronesia, Montserrat, Mozambique, Myanmar, Nauru, Nepal, New Caledonia, New Zealand, Nicaragua, Niger, Nigeria, Oman, Pakistan, Palau, Panama, Papua New Guinea, Paraguay, Peru, Philippines, Puerto Rico, Reunion, Saint Lucia, Saint Vincent and the Grenadines, Samoa, São Tomé-and-Príncipe, Saudi Arabia, Senegal, Singapore, Solomon Islands, South Africa, Sri Lanka, Sudan, South Sudan, Suriname, Tanzania, Thailand, Timor, Togo, Tonga, Trinidad and Tobago, Turkey, Tuvalu, Uganda, USA, Uruguay, Vanuatu, Venezuela, Vietnam, Virgin Islands, Yemen and Zambia [66]. Larvae can be found in water bodies containing a high degree of organic pollution and close to human habitations. Females highly anthropophilic and biting at night [9, 81]. This species is a vector of avian malaria, confirmed vector for Japanese encephalitis virus [6], West Nile virus, St Louis encephalitis virus, Rift valley virus, Edge Hill virus, Eubenangee virus, Getah virus, Kokobera virus, Koongol virus, Kowanyama virus, Kunjin virus, Mapputta virus, Stratford virus, Trubanaman virus, Wongal virus, Chikungunya virus [3]. It is also a primary vector of *W. bancrofti* and it has been implicated as a vector of dog heartworm [81].

###### *Culex* (*Culex*) *sitiens* Wiedemann, 1828

Distribution: Australia, Bangladesh, Cambodia, Cameroon, China, Comoros, Djibouti, Egypt, Ethiopia, Fiji, French Polynesia, Guam, Hong Kong, India, Indonesia, Iran, Japan, Kenya, Koreas, Madagascar, Malaysia, Maldives, Mariana Islands, Morocco, Mozambique, Myanmar, Nauru, New Caledonia, Nigeria, Oman, Oman, Pakistan, Palau, Papua New Guinea, Philippines, Polynesian Islands, Pitcairn, Wallis & Futuna, Samoa, Saudi Arabia, Senegal, Seychelles, Singapore, Solomon Islands, Sri Lanka, Sudan, South Sudan, Taiwan, Tanzania, Thailand, Timor, Tonga, Tuvalu, United Arab Emirates, Vanuatu, Vietnam and Yemen [9, 66]. Larvae are found in brackish, salt and fresh groundwater habitats and some artificial containers in coastal areas. Females feed primarily on birds and pigs, but can bite humans [24]. Possible vector of Japanese encephalitis virus. Found naturally infected with *B. malayi* in Thailand [24].

###### *Culex* (*Culex*) *tritaeniorhynchus* Giles, 1901

Distribution: Afghanistan, Angola, Azerbaijan, Bangladesh, Brunei, Cambodia, Cameroon, Central African Republic, China, Djibouti, Egypt, Ethiopia, Gabon, Gambia, Ghana, Greece, Guam, Hong Kong, India, Indonesia, Iran, Iraq, Israel, Japan, Jordan, Kenya, Koreas, Laos, Lebanon, Liberia, Madagascar, Malaysia, Maldives, Mariana Islands, Mauritius, Micronesia, Mozambique, Myanmar, Nepal, Nigeria, Oman, Pakistan, Papua New Guinea, Philippines, Reunion, Russia, Saudi Arabia, Senegal, Singapore, Somalia, Sri Lanka, Syria, Taiwan, Tanzania, Thailand, Timor, Togo, Turkey, Turkmenistan, USA, Vietnam and Yemen [9, 66]. Larvae are found in many temporary, semi-permanent and permanent groundwater habitats that are sunlit and contain vegetation. Habitats can be ground pools, streams, swamps, and low-salinity tidal marshes [7]. Females are primarily cattle- and pig-biters, but in their absence, they will feed on humans [7]. Species of medical importance, confirmed vector for Japanese encephalitis virus [6].

###### *Culex* (*Culex*) *vishnui* Theobald, 1901

Distribution: Bangladesh, Cambodia, China, Hong Kong, India, Indonesia, Japan, Korea, Laos, Malaysia, Maldives, Myanmar, Nepal, Pakistan, Philippines, Singapore, Sri Lanka, Taiwan, Thailand, Timor and Vietnam [66]. Larvae are typically found in ground pool habitats that include puddles, ditches, ponds, animal and wheel tracks, and rice fields containing emergent and aquatic vegetation. Females feed primarily on pigs and birds, but in their absence will readily bite humans [81]. Important vector of Japanese encephalitis virus [81]. During our sampling in Cambodia from 2016 to 2020, using BG and light traps, this species was the most dominant, representing over 57% of all mosquitoes collected.

###### *Culex* (*Culex*) *whitei* Barraud, 1923

Distribution: Bangladesh, Cambodia, India, Indonesia, Malaysia, Nepal, Philippines, Thailand and Vietnam [66].

###### *Culex* (*Culex*) *whitmorei* (Giles, 1904). New record for Cambodia

Distribution: Australia, Bangladesh, China, India, Indonesia, Japan, Korea, Laos, Malaysia, Nepal, Pakistan, Papua New Guinea, Philippines, Russia, Sri Lanka, Taiwan, Thailand and Vietnam [66]. Species collected throughout Cambodia.

##### Subgenus *Culiciomyia* Theobald, 1907 (9 spp.)

###### *Culex* (*Culiciomyia*) *bailyi* Barraud, 1934

Distribution: Cambodia, India, Indonesia, Malaysia, Myanmar, Papua New Guinea, Philippines, Sri Lanka and Thailand [66].

###### *Culex* (*Culiciomyia*) *fragilis* Ludlow, 1903

Distribution: Cambodia, India, Indonesia, Malaysia, Papua New Guinea, Philippines, Solomon Islands, Sri Lanka, Thailand and Vietnam [66].

###### *Culex* (*Culiciomyia*) *nigropunctatus* Edwards, 1926

Distribution: Bangladesh, Cambodia, China, Hong Kong, India, Indonesia, Japan, Laos, Malaysia, Micronesia, Nepal, Palau, Philippines, Singapore, Sri Lanka, Taiwan, Thailand and Vietnam [66].

###### *Culex* (*Culiciomyia*) *pallidothorax* Theobald, 1905

Male and pupae unknown. Distribution: Bangladesh, Cambodia, China, Hong Kong, India, Indonesia, Japan, Macau, Malaysia, Myanmar, Nepal, Philippines, Sri Lanka, Taiwan, Thailand, Timor and Vietnam [66].

###### *Culex* (*Culiciomyia*) *papuensis* (Taylor, 1914)

Distribution: Borneo, Cambodia, Indonesia, Malaysia, Papua New Guinea, Philippines, Solomon Islands and Thailand [66].

###### *Culex* (*Culiciomyia*) *scanloni* Bram, 1967

Distribution: Cambodia, Indonesia, Malaysia, Philippines, Sri Lanka, Thailand and Vietnam [66].

###### *Culex* (*Culiciomyia*) *spathifurca* (Edwards, 1915)

Distribution: Cambodia, India, Indonesia, Malaysia, Maldives, Philippines, Singapore, Sri Lanka and Thailand [66].

###### *Culex* (*Culiciomyia*) *termi* Thurman, 1955

Distribution: Cambodia and Thailand [66].

###### *Culex* (*Culiciomyia*) *thurmanorum* Bram, 1967

Distribution: Cambodia and Thailand [66].

##### Subgenus *Eumelanomyia* Theobald, 1909 (11 spp.)

###### *Culex* (*Eumelanomyia*) *bokorensis* Klein & Sirivanakarn, 1970

Female and larval stages unknown. Distribution: species only known from Cambodia [31].

###### *Culex* (*Eumelanomyia*) *brevipalpis* (Giles, 1902)

Distribution: Bangladesh, Cambodia, China, India, Indonesia, Japan, Malaysia, Myanmar, Nepal, Pakistan, Papua New Guinea, Philippines, Singapore, Sri Lanka, Taiwan, Thailand and Vietnam [66].

###### *Culex* (*Eumelanomyia*) *foliatus* Brug, 1932

Distribution: Cambodia, China, Hong Kong, India, Indonesia, Malaysia, Nepal, Philippines, Sri Lanka, Taiwan, Thailand, Timor and Vietnam [66].

###### *Culex* (*Eumelanomyia*) *hinglungensis* Chu, 1957

Distribution: Cambodia, China, India, Philippines and Thailand [66].

###### *Culex* (*Eumelanomyia*) *kiriensis* Klein & Sirivanakarn, 1970

Distribution: Cambodia and Thailand [31, 66].

###### *Culex* (*Eumelanomyia*) *malayi* (Leicester, 1908)

Distribution: Bangladesh, Cambodia, China, India, Indonesia, Malaysia, Maldives, Myanmar, Nepal, Pakistan, Sri Lanka, Taiwan, Thailand, Timor and Vietnam [66].

###### *Culex* (*Eumelanomyia*) *oresbius* Harbach & Rattanarithikul, 1988

Distribution: Cambodia and Thailand [66].

###### *Culex* (*Eumelanomyia*) *otachati* Klein & Sirivanakarn, 1970

Pupae unknown. Distribution: Cambodia and Thailand [66].

###### *Culex* (*Eumelanomyia*) *richei* Klein, 1970

Pupae unknown. Distribution: Cambodia and Thailand [66].

###### *Culex* (*Eumelanomyia*) *selai* Klein & Sirivanakarn, 1970

Female unknown. Distribution: Cambodia and Malaysia [82].

###### *Culex* (*Eumelanomyia*) *tenuipalpis* Barraud, 1924

Distribution: Cambodia, India, Indonesia, Malaysia and Thailand [66].

##### Subgenus *Lophoceraomyia* Theobald, 1905 (18 spp.)

###### *Culex* (*Lophoceraomyia*) *aculeatus* Colless, 1965

Distribution: Cambodia, Malaysia and Thailand [66].

###### *Culex* (*Lophoceraomyia*) *cinctellus* Edwards, 1922

Distribution: Cambodia, China, India, Indonesia, Japan, Malaysia, Philippines, Singapore, Thailand and Vietnam [66].

###### *Culex* (*Lophoceraomyia*) *curtipalpis* (Edwards, 1914)

Distribution: Cambodia, Indonesia, Malaysia, Singapore, Thailand and Vietnam [66].

###### *Culex* (*Lophoceraomyia*) *eukrines* Bram & Rattanarithikul, 1967

Distribution: Cambodia and Thailand [66].

###### *Culex* (*Lophoceraomyia*) *fraudatrix* (Theobald, 1905). New record for Cambodia

Distribution: Australia, Indonesia, Malaysia, New Caledonia, Papua New Guinea, Philippines, Solomon Islands and Thailand [81]. Collected in Kampong Thom province.

###### *Culex* (*Lophoceraomyia*) *ganapathi* Colless, 1965

Distribution: Cambodia, Malaysia and Thailand [66].

###### *Culex* (*Lophoceraomyia*) *inculus* Colless, 1965

Distribution: Cambodia, Indonesia and Malaysia [81].

###### *Culex* (*Lophoceraomyia*) *infantulus* Edwards, 1922

Distribution: Cambodia, China, Hong Kong, India, Indonesia, Japan, Korea, Malaysia, Maldives, Myanmar, Nepal, Philippines, Sri Lanka, Thailand and Vietnam [66].

###### *Culex* (*Lophoceraomyia*) *macdonaldi* Colless, 1965

Distribution: Cambodia, India, Indonesia, Malaysia, Philippines, Singapore, Thailand and Vietnam [66].

###### *Culex* (*Lophoceraomyia*) *mammilifer* (Leicester, 1908)

Distribution: Cambodia, China, India, Indonesia, Malaysia, Philippines, Sri Lanka and Thailand [66].

###### *Culex* (*Lophoceraomyia*) *minor* (Leicester, 1908)

Distribution: Cambodia, China, India, Indonesia, Malaysia, Philippines, Thailand and Vietnam [66].

###### *Culex* (*Lophoceraomyia*) *peytoni* Bram & Rattanarithikul, 1967

Distribution: Cambodia, India, Indonesia, Malaysia, Thailand and Vietnam [66].

###### *Culex* (*Lophoceraomyia*) *quadripalpis* (Edwards, 1914)

Distribution: Cambodia, India, Indonesia, Malaysia, Philippines, Singapore, Sri Lanka, Thailand and Vietnam [66].

###### *Culex* (*Lophoceraomyia*) *reidi* Colless, 1965

Distribution: Cambodia, Indonesia, Malaysia, Philippines, Singapore and Thailand [66].

###### *Culex* (*Lophoceraomyia*) *rubithoracis* (Leicester, 1908)

Distribution: Cambodia, China, Guam, Hong Kong, India, Indonesia, Japan, Malaysia, Myanmar, Philippines, Singapore, Sri Lanka, Taiwan, Thailand and Vietnam [66].

###### *Culex* (*Lophoceraomyia*) *spiculosus* Bram & Rattanarithikul, 1967

Distribution: Cambodia, Indonesia, Malaysia, Myanmar, Taiwan and Thailand [66].

###### *Culex* (*Lophoceraomyia*) *variatus* (Leicester, 1908)

Distribution: Cambodia, China, India, Indonesia, Malaysia, Singapore, Thailand and Vietnam [66].

###### *Culex* (*Lophoceraomyia*) *wilfredi* Colless, 1965

Distribution: Cambodia, China, India, Malaysia, Thailand and Vietnam [66].

##### Subgenus *Oculeomyia* Theobald, 1907 (3 spp.)

###### *Culex* (*Oculeomyia*) *bitaeniorhynchus* Giles, 1901

Distribution: Angola, Australia, Bangladesh, Benin, Burkina Faso, Cambodia, Cameroon, Cape Verde, China, Democratic Republic of the Congo, Djibouti, Egypt, Gabon, Gambia, Ghana, Hong Kong, India, Indonesia, Iran, Japan, Kenya, Korea, Laos, Lesotho, Liberia, Madagascar, Malaysia, Micronesia, Mozambique, Myanmar, Namibia, Nepal, New Caledonia, Nigeria, Pakistan, Palau, Papua New Guinea, Philippines, Russia, Saudi Arabia, Senegal, Singapore, Solomon Islands, Somalia, South Africa, Sri Lanka, Sudan, South Sudan, Taiwan, Tanzania, Thailand, Uganda, Vietnam, Yemen, Zambia and Zimbabwe [64]. Immature stages restricted to groundwater habitats containing *Spirogyra* algae. Females are primarily bird feeders in Thailand. In New Guinea, a form readily bites humans [7, 24]. Found naturally infected with *Wuchereria bancrofti* in India, and *Brugia malayi* in Sri Lanka [24].

###### *Culex* (*Oculeomyia*) *pseudosinensis* Colless, 1955

Distribution: Cambodia, Indonesia, Laos, Malaysia, Singapore, Thailand and Vietnam [66].

###### *Culex* (*Oculeomyia*) *sinensis* Theobald, 1903

Distribution: Australia, Bangladesh, Cambodia, China, Hong Kong, India, Indonesia, Japan, Korea, Laos, Malaysia, Myanmar, Nepal, Papua New Guinea, Philippines, Russia, Sri Lanka, Sudan, South Sudan, Taiwan, Thailand and Vietnam [62].

#### Genus *Culiseta* Felt, 1904 (1 sp.)

The genus *Culiseta* includes 39 species divided among 7 subgenera (*Allotheobaldia, Austrotheobaldia, Climacura, Culicella, Culiseta, Neotheobaldia,* and *Theomyia*) [25, 85]. The *Culiseta* are large mosquitoes and cold-adapted species which occur only in warmer climates during the colder times of the year or at higher elevations where temperatures are low. Only the subgenus *Culimacura* is present in Cambodia with one species. Immature stages of *Culiseta* (*Climacura*) species are usually found in permanent bodies of water, mainly swamps, but may also inhabit semi-permanent pools. Females primarily feed on birds but can occasionally feed on humans.

##### Subgenus *Climacura* Howard, Dyar & Knab, 1915 (1 sp.)

###### *Culiseta* (*Climacura*) *marchettei* Garcia, Jeffery & Rudnick, 1969

Distribution: Cambodia and Malaysia [18].

#### Genus *Lutzia* Theobald, 1903 (3 spp.)

The genus *Lutzia* includes 9 species divided among 3 subgenera: *Insulalutzia*, *Lutzia*, and *Metalutzia* [25]. Only the latter is represented in Cambodia by 3 species. Adults are large, and females feed on livestock, and rarely on humans. Very little information is known about the biology of the adults. Larvae are predaceous and occur in a wide variety of groundwater habitats, from tree holes to artificial cavities. Generally, they appreciate water with high organic content [25].

##### Subgenus *Metalutzia* Tanaka, 2003 (3 spp.)

###### *Lutzia* (*Metalutzia*) *fuscana* (Wiedemann, 1820)

Distribution: Bangladesh, Cambodia, China, India, Indonesia, Japan, Korea, Malaysia, Mariana Islands, Micronesia, Myanmar, Nepal, Pakistan, Palau, Philippines, Russia, Singapore, Sri Lanka, Taiwan, Thailand, Timor and Vietnam [66]. Upon hatching, the larvae will immediately consume nearby, similar-sized, larvae. Adult female seems to feed preferentially on avian hosts [7].

###### *Lutzia* (*Metalutzia*) *halifaxii* (Theobald, 1903)

Distribution: Australia, Bangladesh, Cambodia, China, India, Indonesia, Japan, Koreas, Malaysia, Nepal, Pakistan, Papua New Guinea, Philippines, Russia, Singapore, Solomon Islands, Sri Lanka, Taiwan, Thailand, Timor and Vietnam [66]. The larvae can be found in various kinds of temporary and semipermanent groundwater habitats and occur frequently in artificial containers as well as rock pools, stream margins and tree cavities [7]. The larvae prefer water containing high organic content. They are predaceous and can attack other arthropods nearby. The host preference for the adult female is not known, but humans have been reported to be an occasional host [7].

###### *Lutzia* (*Metalutzia*) *vorax* Edwards, 1921. New record for Cambodia

Distribution: Bhutan, China, India, Indonesia, Japan, Malaysia, Mariana Islands, Myanmar, Nepal, South-Korea, Sri Lanka, Taiwan and Thailand [56, 66]. Collected in Kampong Cham and Battambang provinces.

#### Genus *Ficalbia* Theobald, 1903 (1 sp.)

The genus *Ficalbia* is represented by 8 species present in the Afrotropical, Palearctic and Oriental regions [25]. Only one species is present in Cambodia. Very little is known about the biology of this genus. Their larvae can be found in swamps, marshes, ponds, pools and river margins with abundant vegetation. Nothing is known about the biting habits of females [67].

##### 

###### *Ficalbia minima* (Theobald, 1901)

Distribution: Australia, Bangladesh, Cambodia, China, India, Indonesia, Laos, Malaysia, Myanmar, Papua New Guinea, Singapore, Sri Lanka, Thailand and Vietnam [67]. Collected in Cambodia in primary forests, in shaded areas.

#### Genus *Mimomyia* Theobald, 1903 (7 spp.)

The genus *Mimomyia* includes 45 species shared among 3 subgenera: *Etorleptiomyia, Ingramia,* and *Mimomyia* [25]. The 3 subgenera are present in Cambodia hosting 2, 2 and 3 species, respectively. *Mimomyia* are generally small mosquitoes. Very little is known about the biology of this genus. The larvae of most species occur in swamps and marshes with dense vegetation. The larval siphon of several species is modified for piercing aquatic plants to obtain oxygen. Larvae of subgenus *Ingramia* are found in the leaf axils of plants. The adults of several species have been reported to bite humans, but none are serious pests. Most species appear to be active at night.

##### Subgenus *Etorleptiomyia* Theobald, 1904 (2 spp.)

###### *Mimomyia* (*Etorleptiomyia*) *elegans* (Taylor, 1914)

Distribution: Australia, Cambodia, Indonesia, Japan, Malaysia, Papua New Guinea, Philippines, Solomon Islands, Thailand, USA and Vietnam [67].

###### *Mimomyia* (*Etorleptiomyia*) *luzonensis* (Ludlow, 1905)

Distribution: Cambodia, China, Hong Kong, India, Indonesia, Japan, Malaysia, Myanmar, Nepal, Pakistan, Philippines, Singapore, Sri Lanka, Taiwan, Thailand and Vietnam [67].

##### Subgenus *Ingramia* Edwards, 1912 (2 spp.)

###### *Mimomyia* (*Ingramia*) *fusca* (Leicester, 1908)

Distribution: Cambodia, Indonesia, Malaysia, Philippines, Singapore, Taiwan and Thailand [67].

###### *Mimomyia* (*Ingramia*) *kiriromi* (Klein, 1969)

Immature stages unknown. Distribution: known only from Cambodia [25].

##### Subgenus *Mimomyia* Theobald, 1904 (3 spp.)

###### *Mimomyia* (*Mimomyia*) *aurea* (Leicester, 1908)

Distribution: Cambodia, India, Malaysia, Singapore, Thailand and Vietnam [67].

###### *Mimomyia* (*Mimomyia*) *chamberlaini* Ludlow, 1904

Distribution: Australia, Bangladesh, Cambodia, China, India, Indonesia, Malaysia, Myanmar, Nepal, Papua New Guinea, Philippines, Sri Lanka, Thailand and Vietnam [67]. Collected in Cambodia only from Battambang province using BG traps.

###### *Mimomyia* (*Mimomyia*) *hybrida* (Leicester, 1908)

Distribution: Bangladesh, Cambodia, India, Indonesia, Laos, Malaysia, Myanmar, Nepal, Philippines, Singapore, Sri Lanka, Thailand and Vietnam [67].

#### Genus Hodgesia Theobald, 1904 (3 spp.)

The genus *Hodgesia* consists of 11 species [25]. These mosquitoes are minute in size, and the immature stages can be found in swamps and marshes, in water containing very dense vegetation, often associated with larvae of *Mimomyia spp*. The feeding habits of most species are unknown, but some species have been reported to bite humans [25].

##### Subgenus *Hodgesia* Theobald, 1904 (3 spp.)

###### *Hodgesia* (*Hodgesia*) *bailyi* Barraud, 1929

Distribution: Cambodia, India, Sri Lanka, Thailand and Vietnam [67].

###### *Hodgesia* (*Hodgesia*) *malayi* Leicester, 1908

Distribution: Cambodia, Indonesia, Laos, Malaysia, Philippines, Sri Lanka, Thailand and Vietnam [67].

###### *Hodgesia* (*Hodgesia*) *quasisanguinae* Leicester, 1908

Distribution: Australia, Cambodia, Indonesia, Malaysia, Papua New Guinea and Philippines [67].

#### Genus *Coquillettidia* Dyar, 1905 (3 spp.)

The genus *Coquillettidia* consists of 58 species, generally large, and yellowish [25]. The genus is subdivided into 3 subgenera: *Austromansonia*, *Rhynchotaenia,* and *Coquillettidia* [25]. Only the latter is present in Cambodia and comprises 3 species. Their larvae attach themselves to aquatic plants to obtain oxygen. Adult females are known to bite humans and domestic animals [59].

##### Subgenus *Coquillettidia* Dyar, 1905 (3 spp.)

###### *Coquillettidia* (*Coquillettidia*) *crassipes* (van der Wulp, 1881). New record for Cambodia

Distribution: China, Hong Kong, India, Indonesia, Macau, Malaysia, Mariana Island, Myanmar, Pakistan, Papua New Guinea, Philippines, Sri Lanka and Thailand [59]. Collected in Koh Kong, Rattanak Kiri, Kampong Cham, Kampong Saom, Preah Vihear, Pursat and Battambang provinces.

###### *Coquillettidia* (*Coquillettidia*) *nigrosignata* (Edwards, 1917)

Distribution: Cambodia, Indonesia, Malaysia, Philippines, Singapore, Taiwan and Thailand [30, 67].

###### *Coquillettidia* (*Coquillettidia*) *ochracea* (Theobald, 1903)

Distribution: Bangladesh, Cambodia, China, India, Indonesia, Japan, Korea, Laos, Malaysia, Papua New Guinea, Philippines, Thailand and Vietnam [67].

#### Genus Mansonia Blanchard, 1901 (5 spp.)

The genus *Mansonia* includes 25 species subdivided into 2 subgenera [25]. The subgenus *Mansonia* comprises 15 species distributed in the New World, while *Mansonioides* occurs in the Old World and consists of 10 species. Only the latter is present in Cambodia with 5 species. The larvae of *Mansonia* occur in permanent waters in association with aquatic plants and attach themselves to their floating roots in order to obtain oxygen. Females of several species are nocturnal, and are known to transmit several arboviruses [25].

##### Subgenus *Mansonioides* Theobald, 1907 (5 spp.)

###### *Mansonia* (*Mansonioides*) *annulifera* (Theobald, 1901). New record for Cambodia

Distribution: India, Myanmar, Sri Lanka, Malaysia, Thailand and Philippines. Known to transmit Chikungunya [104]. Species collected in Battambang, Kampong Cham, Preah Vihear and Pursat provinces.

###### *Mansonia* (*Mansonioides*) *bonneae* Edwards, 1930

Distribution: Cambodia, Indonesia, Malaysia, Philippines, Singapore and Thailand [67].

###### *Mansonia* (*Mansonioides*) *dives* (Schiner, 1868)

Distribution: Bangladesh, Cambodia, China, India, Indonesia, Malaysia, Philippines, Singapore, Thailand and Vietnam [67]. Immature stages can be found in shaded pools in swamp forest. Adults are crepuscular and females seem to prefer cattle but can feed on humans [102].

###### *Mansonia* (*Mansonioides*) *indiana* Edwards, 1930

Distribution: Cambodia, India, Indonesia, Laos, Malaysia, Myanmar, Nepal, Philippines, Singapore, Sri Lanka, Thailand and Vietnam [67].

###### *Mansonia* (*Mansonioides*) *uniformis* (Theobald, 1901)

Distribution: Angola, Australia, Bangladesh, Benin, Botswana, Burkina Faso, Cambodia, Central African Republic, China, Comoros, Cote d’Ivoire, Ethiopia, Gabon, Gambia, Ghana, Guam, Hong Kong, India, Indonesia, Japan, Kenya, South Korea, Liberia, Madagascar, Malawi, Malaysia, Mali, Mozambique, Myanmar, Nepal, Niger, Nigeria, Pakistan, Papua New Guinea, Philippines, Senegal, Sierra Leone, Solomon Islands, South Africa, Sri Lanka, Sudan, Taiwan, Tanzania, Thailand, Timor, Uganda, Vietnam and Zambia. Immature stages can be found in swamps and pools containing abundant vegetation [9, 54, 67].

#### Genus *Orthopodomyia* Theobald, 1904 (3 spp.)

The genus *Orthopodomyia* consist of 36 species distributed worldwide. Very little is known about their biology. Larvae occur principally in tree holes, but some species are found in bamboo internodes, inside Bromeliaceae and *Heliconia* plants. The larvae can sometimes be found in artificial containers. Adults are nocturnal and are found mainly in forests. The feeding habits of females are largely unknown, but they are probably ornithophilic. Two species in the Oriental Region are known to approach and bite humans. This genus is not considered to be of medical importance [25].

##### 

###### *Orthopodomyia* (*Orthopodomyia*) *andamanensis* Barraud, 1934

Distribution: Andaman Islands, Cambodia, Indonesia, Malaysia, Philippines, Thailand and Vietnam [65]. The immature stages have been observed to breed in tree holes, bamboo stumps and artificial containers [110]. In Cambodia, the larvae were collected in artificial containers.

###### *Orthopodomyia* (*Orthopodomyia*) *anopheloides* (Giles, 1903)

Distribution: Cambodia, China, India, Indonesia, Japan, Malaysia, Nepal, Pakistan, Philippines, Singapore, Sri Lanka, Taiwan, Thailand and Vietnam [65]. The immature stages have been observed in tree holes, bamboo stumps and artificial containers [110].

###### *Orthopodomyia* (*Orthopodomyia*) *siamensis* Zavortink, 1968. New record for Cambodia

Distribution: this species was only known from Thailand [65]. Species collected in Mondul Kiri and Rattanak Kiri provinces.

#### Genus *Malaya* Leicester, 1908 (2 spp.)

##### 

The genus *Malaya* is a small group represented by only 12 species [25]. The immature stages can be found in plant cavities, such as leaf axils, tree holes or water-filled nests of arboreal ants. The adults are diurnal and incapable of taking a blood meal. Their feeding habits are unique among mosquitoes: they feed on the regurgitation of ants: specifically, both sexes obtain a sugar meal from ants that have collected honeydew from scale insects (Hemiptera: Coccoidea). To accomplish this, the mosquito accosts an ant and brings the tip of its proboscis into contract with the mouth of the ant until a drop of liquid is produced. The regurgitated liquid is rapidly sucked up and the ant goes away unharmed. This genus is represented by two species in Cambodia.

###### *Malaya* (*Malaya*) *genurostris* Leicester, 1908

Distribution: Australia, India, Indonesia, Japan, Malaysia, Maldives, Myanmar, Papua New Guinea, Philippines, South China, Singapore, Taiwan, Thailand and Sri Lanka, [44].

###### *Malaya* (*Malaya*) *jacobsoni* (Edwards, 1930)

Distribution: Bangladesh, Cambodia, India, Indonesia, Malaysia, Nepal, Taiwan, Thailand and Vietnam [67].

#### Genus *Topomyia* Leicester, 1908 (4 spp.)

The genus *Topomyia* includes 65 species in 2 subgenera: *Miyagiella* and *Topomyia*. Only the latter is present in Cambodia represented with 4 species. *Topomyia* are considered forest mosquitoes. Very little is known about the biology of the adults, but as females do not feed on humans they are not considered to be of medical importance. The larvae can be found in leaf axils, *Nepenthes* pitcher plants, bamboo internodes and stumps and sometimes tree holes [25].

##### Subgenus *Topomyia* Leicester, 1908 (4 spp.)

###### *Topomyia* (*Topomyia*) *angkoris* Klein, 1977

Distribution: Cambodia and Thailand [34, 65].

###### *Topomyia* (*Topomyia*) *apsarae* Klein, 1977

Described from Cambodia [34], recorded from Philippines (Palawan islands). According to Miyagi et al. [43], this species has very particular habits: the larvae were only collected in bamboo internodes bearing a tiny hole bored by a beetle. The larvae preys upon small crustacean and chironomid larvae.

###### *Topomyia* (*Topomyia*) *argyropalpis* Leicester, 1908

Distribution: Cambodia, Indonesia, Malaysia, Philippines and Thailand [65].

###### *Topomyia* (*Topomyia*) *gracilis* Leicester, 1908

Distribution: Malaysia, Indonesia, Thailand, Vietnam, Laos and Cambodia. Larvae can be found in leaf axils of plants [45, 88].

#### Genus *Tripteroides* Giles, 1904 (6 spp.)

The genus *Tripteroides* hosts 122 species divided into five subgenera: *Polylepidomyia*, *Rachionotomyia*, *Rachisoura*, *Tricholeptomyia* and *Tripteroides.* Only 2 (*Rachionotomyia* and *Tripteroides*) are present in Cambodia. The larvae inhabit small collections of water in tree holes, bamboo, coconut shells and husks, fallen leaves, pitcher plants or even snail shells. They feed on arthropods or their remains. Adults are diurnal. A few species feed on humans, but nothing is known about the feeding habits of most species [25].

##### Subgenus *Rachionotomyia* Theobald, 1905 (2 spp.)

###### *Tripteroides* (*Rachionotomyia*) *affinis* (Edwards, 1913). New record for Cambodia

Distribution: India, Sri Lanka and Thailand [65]. Collected in Cambodia from larvae reared from *Nepenthes* water, collected in the primary forest of Rattanak Kiri and Mondul Kiri provinces.

###### *Tripteroides* (*Rachionotomyia*) *aranoides* (Theobald, 1901)

Distribution: Bangladesh, Cambodia, China, India, Indonesia, Laos, Malaysia, Myanmar, Nepal, Singapore, Sri Lanka, Taiwan, Thailand and Vietnam [65]. The larvae can be found in bamboo, tree holes, pitcher plants [96]. Among the Oriental species, only *Tr. aranoides* is known to bite humans [25].

##### Subgenus *Tripteroides* Giles, 1904 (4 spp.)

###### *Tripteroides* (*Tripteroides*) *aeneus* (Edwards, 1921)

Distribution: Cambodia, Malaysia and Thailand [65]. Collected in Cambodia from clear water in tree holes, in the primary forest of Mondul Kiri province.

###### *Tripteroides* (*Tripteroides*) *caeruleocephalus* (Leicester, 1908). New record for Cambodia

Distribution: Indonesia, Malaysia and Thailand [65]. Collected in Pursat province.

###### *Tripteroides* (*Tripteroides*) *powelli* (Ludlow, 1909)

Distribution: Cambodia, China, India, Indonesia, Malaysia, Philippines, Thailand and Vietnam [65]. Species found in bamboo forests [96].

###### *Tripteroides* (*Tripteroides*) *similis* (Leicester, 1908)

Distribution: Cambodia, China, India, Indonesia, Malaysia and Thailand [65]. Species found in bamboo forests [96].

#### Genus *Toxorhynchites* Theobald, 1901 (4 spp.)

The genus *Toxorhynchites* comprises 89 species worldwide, divided into 4 subgenera: *Afrorhynchus, Ankylorhynchus, Lynchiella* and *Toxorhynchites* [25]. The Cambodian species belong exclusively to the subgenus *Toxorhynchites*. The larvae are predators, feeding mainly on other mosquito larvae. They can be found in plant cavities, mainly tree holes and bamboo stumps [95]. Adults are covered with iridescent scales and are typically large and colorful mosquitoes. Both males and females are diurnal and feed exclusively on nectar and other sugary substances. They do not display blood-sucking behavior and are not considered to be of medical importance, but their larvae can be used for pest control [14, 87].

##### Subgenus *Toxorhynchites* Theobald, 1901 (4 spp.)

###### *Toxorhynchites* (*Toxorhynchites*) *albipes* (Edwards, 1922)

Distribution: Cambodia, India, Laos, Thailand and Vietnam [65, 99].

###### *Toxorhynchites* (*Toxorhynchites*) *gravelyi* (Edwards, 1921). New record for Cambodia

Distribution: India, Malaysia and Thailand [99]. Specimens obtained from larvae collected in tree holes in Mondul Kiri province.

###### *Toxorhynchites* (*Toxorhynchites*) *kempi* (Edwards, 1921)

Distribution: Cambodia, India, Indonesia, Laos, Philippines and Vietnam [65].

###### *Toxorhynchites* (*Toxorhynchites*) *splendens* (Wiedemann, 1819)

Distribution: Australia, Bangladesh, Cambodia, China, Fiji, India, Indonesia, Malaysia, Myanmar, Nepal, Papua New Guinea, Philippines, Sri Lanka, Thailand and Vietnam [65].

#### Genus *Uranotaenia* Lynch Arribálzaga, 1891 (27 spp.)

This genus includes 271 species worldwide divided into 2 subgenera: *Pseudoficalbia* and *Uranotaenia*. Both are present in Cambodia, hosting 13 and 14 species, respectively [25]. The feeding preferences of most species are currently unknown but field observation tends to indicate that amphibians, reptiles, birds and mammals serve as hosts. Females can bite humans but do not seem to be involved in pathogen transmission. Immature stages can be found in a wide range of habitats: most species inhabit groundwaters, including swamps, marshes, stream margins and temporary pools with vegetation, but many also use crab holes, tree holes, bamboo, plant parts on the ground, leaf axils, flower bracts, pitcher plants or artificial containers [25].

##### Subgenus *Pseudoficalbia* Theobald, 191*2* (13 spp.)

###### *Uranotaenia* (*Pseudoficalbia*) *abdita* Peyton, 1977

Distribution: Cambodia and Thailand [55]. Immature stages seem to be associated with crab holes, and occur essentially in small freshwater crab holes at the margin of shallow running mountain or foothill streams, or at the edge of springs or seepages and generally under the cover of secondary or primary forests. Adults can be observed resting on rocks over streams, up to an elevation of 1000 m [55].

###### *Uranotaenia* (*Pseudoficalbia*) *albipes* Peyton, 1977. New record for Cambodia

Male unknown. Distribution: Thailand [67]. Collected in Rattanak Kiri province only.

###### *Uranotaenia* (*Pseudoficalbia*) *bicolor* Leicester, 1908

Distribution: Cambodia, China, India, Indonesia, Malaysia, Nepal, Philippines, Sri Lanka, Thailand and Vietnam [55, 67]. According to Peyton [55], this species is the most common of the genus in Southeast Asia, immature stages tend to colonize a very wide range of aquatic habitats. While it is present to an elevation up to 1000 m, most of the collection (95%) is below 350 m.

###### *Uranotaenia* (*Pseudoficalbia*) *gouldi* Peyton & Klein, 1970

Distribution: Thailand and Cambodia [55]. Immature stages can be found in swamps, seepage pool or bog, or stream pool [67].

###### *Uranotaenia* (*Pseudoficalbia*) *hirsutifemora* Peters, 1964

Distribution: Australia, Cambodia, Indonesia, Malaysia, Papua New Guinea, Singapore, Solomon Islands and Thailand [67]. Most of the collection of immature stages was done in crab holes and marsh swamps areas [55].

###### *Uranotaenia* (*Pseudoficalbia*) *koli* Peyton & Klein, 1970

Distribution: Cambodia, Thailand and Vietnam [55]. Species restricted to forested hills and mountainous areas. Immature stages can be collected in crab holes on the banks of shallow fresh running streams and elephant footprints in bogs where crab holes were present. Adult often rest on vegetation or rocks along stream margins [55].

###### *Uranotaenia* (*Pseudoficalbia*) *lutescens* Leicester, 1908

Distribution: Cambodia, India, Malaysia, Thailand and Vietnam [55, 67]. Prefer secondary forests where bamboo habitats are abundant. Immature stages show a preference for a variety of bamboo habitats, located on or near the ground, bamboo internodes, with small or moderate entrance holes, bamboo stumps, cut bamboo, or tree stumps. Most collections were done at an elevation below 600 m [55]. Collected in Cambodia from primary forest in Mondul Kiri province, the immature stages were collected from transparent water colored with tannin in bamboo tree holes.

###### *Uranotaenia* (*Pseudoficalbia*) *maxima* Leicester, 1908. New record for Cambodia

Distribution: China, India, Malaysia and Thailand [67]. Collected in Mondul Kiri and Kampong Thom provinces.

###### *Uranotaenia* (*Pseudoficalbia*) *nivipleura* Leicester, 1908

Distribution: Cambodia, China, Hong Kong, India, Indonesia, Japan, Laos, Malaysia, Nepal, Singapore, Sri Lanka, Taiwan, Thailand and Vietnam [55, 67]. Immature stages collected from tree stumps tree holes and artificial containers. Mainly found in forests and anthropic environments at elevations up to 2286 m [55].

###### *Uranotaenia* (*Pseudoficalbia*) *novobscura* Barraud, 1934

Distribution: Bangladesh, Cambodia, China, Hong Kong, India, Japan, Laos, Malaysia, Taiwan and Thailand [55, 67]. Immature stages usually found in bamboo stumps, tree holes and sometimes artificial containers. Immature stages are usually associated with larvae of *Aedes sp., Tripteroides sp.* or *Culex sp.* [55]. Adults do not bite humans but seems to feed on toads [55].

###### *Uranotaenia* (*Pseudoficalbia*) *obscura* Edwards, 1915

Distribution: Australia, Cambodia, India, Indonesia, Malaysia, Papua New Guinea, Philippines, Singapore, Sri Lanka and Thailand [55, 67]. Immature stages can colonize a variety of habitats, usually containing a small amount of water, including both natural and artificial containers, often found associated with *Aedes*, *Armigeres*, *Culex* and *Zeugnomyia* larvae [55].

###### *Uranotaenia* (*Pseudoficalbia*) *pseudomaculipleura* Peyton & Rattanarithikul, 1970. New record for Cambodia

Distribution: Malaysia and Thailand [67]. Collected in Rattanak Kiri province.

###### *Uranotaenia* (*Pseudoficalbia*) *spiculosa* Peyton & Rattanarithikul, 1970

Distribution: Cambodia, Thailand and Vietnam [55, 67]. Immature stages have been collected in small freshwater crab holes, along the banks of shallow running streams, generally with dense forest cover [55].

##### Subgenus *Uranotaenia* Lynch Arribálzaga, 1891 (14 spp.)

###### *Uranotaenia* (*Uranotaenia*) *annandalei* Barraud, 1926

Distribution: Cambodia, China, India, Japan, Myanmar, Nepal, Philippines, Philippines, Taiwan, Thailand and Vietnam [67]. Immature stages usually associated with shady pools of water [37].

###### *Uranotaenia* (*Uranotaenia*) *bimaculiala* Leicester, 1908

Distribution: Cambodia, India, Indonesia, Japan, Malaysia and Thailand [37, 67]. Adults collected in bamboo forests.

###### *Uranotaenia* (*Uranotaenia*) *campestris* Leicester, 1908

Distribution: Bangladesh, Cambodia, India, Indonesia, Malaysia, Nepal, Sri Lanka, Thailand, Timor and Vietnam [37, 67]. Adults collected near streams and rock springs.

###### *Uranotaenia* (*Uranotaenia*) *diraphati* Peyton & Klein, 1970

Eggs unknown. Distribution: Cambodia, Malaysia and Thailand [54, 67].

###### *Uranotaenia* (*Uranotaenia*) *lateralis* Ludlow, 1905. New record for Cambodia

Distribution: Australia, China, India, Indonesia, Malaysia, Papua New Guinea, Philippines, Solomon Islands, Sri Lanka, Thailand, Timor and Vietnam [67]. Collected in Preah Vihear province.

###### *Uranotaenia* (*Uranotaenia*) *longirostris* Leicester, 1908

Egg and pupa unknown. Distribution: Cambodia, India, Indonesia, Malaysia, Thailand and Vietnam [37, 67]. Immature stages seem to prefer clear pools near streams. Collected in Cambodia only in Rattanak Kiri province, using light traps.

###### *Uranotaenia* (*Uranotaenia*) *macfarlanei* Edwards, 1914

Eggs and male unknown. Distribution: Cambodia, China, Hong Kong, India, Indonesia, Japan, Malaysia, Nepal, Taiwan, Thailand and Vietnam [37, 67]. The three known larvae specimens were collected in a small pool of dirty water around 900 m above sea-level.

###### *Uranotaenia* (*Uranotaenia*) *metatarsata* Edwards, 1914

Distribution: Cambodia, Indonesia, Malaysia, Philippines, Thailand and Vietnam [67]. Collected in Cambodia from the mangrove forest in Koh Kong province using a light trap.

###### *Uranotaenia* (*Uranotaenia*) *micans* Leicester, 1908

Distribution: Cambodia, Indonesia, Malaysia, Thailand and Vietnam [58, 67].

###### *Uranotaenia* (*Uranotaenia*) *rampae* Peyton & Klein, 1970

Eggs unknown. Known from Cambodia, Malaysia, Thailand and Vietnam [67]. Adults and pupae found in partially shaded swamp with abundant vegetation [54].

###### *Uranotaenia* (*Uranotaenia*) *sombooni* Peyton & Klein, 1970

Egg unknown. Distribution: Cambodia, Malaysia and Thailand [54, 67]. Species apparently restricted to forested hill and mountainous areas, breeds commonly in partially to heavily shaded streams and seepage pools with abundant dead leaves and sticks, but could also be collected from rock pools [54].

###### *Uranotaenia* (*Uranotaenia*) *subnormalis* Martini, 1920

Distribution: Cambodia, Indonesia, Malaysia, Singapore, Thailand and Vietnam [67].

###### *Uranotaenia* (*Uranotaenia*) *testacea* Theobald, 1905

Eggs unknown. Distribution: Cambodia, China, India, Malaysia, Myanmar, Nepal, Singapore and Thailand [67].

###### *Uranotaenia* (*Uranotaenia*) *trilineata* Leicester, 1908

Distribution: Cambodia, Malaysia and Thailand [67].

## Discussion

### Diversity of the Cambodian Culicidae fauna

Between 2016 and 2020, the medical and veterinary entomology of IPC collected more than 230,000 mosquitoes belonging to 193 species of mosquitoes from 16 genera. This unprecedented sampling and identification effort allowed us to increase previous estimates from 241 species [25, 32] to 290 species. We focused the collecting efforts on 11 provinces with diverse ecological settings and biotopes, namely Battambang, Kampong Cham, Kompong Thom, Kompong Saom, Koh Kong, Mondul Kiri, Phnom Penh, Pursat, Preah Viehar, Rattanak Kiri and Siem Reap. However, this sampling is only partial: the country comprises 24 provinces [79] and some of them have a great biological potential. But due to past conflicts, land mine presence or just accessibility, some regions have been under – if not at all – studied over the past decades. Consequently, we can expect many more species to be added to the list in the future. An important limitation of our work is that the identifications were done only morphologically on adults, mostly females. It is certain that species complexes also exist outside the genus *Anopheles*. The probable sibling species, for example in the genera *Culex* or *Aedes*, remain to be discovered.

As a comparison, the fauna of Thailand, with which Cambodia shares a similar fauna, comprises 174 more species (*n* = 464) than Cambodia [64], highlighting the fact that many new records, and probably new species are expected to be described. Further collecting efforts should concentrate on areas like the Cardamone, Kampot, or the Aoral mountains, and the forests in Rattanak Kiri and Mondul Kiri hosting some of the most biologically diverse biomes in the country [48, 79].

However, despite our collection efforts, some species previously listed, only from Cambodia, were not found. This is the case for *Aedes* (*Bothaella*) *kleini* Reinert, 1973, collected in Kampong Speu, *Verrallina* (*Harbachius*) *stunga* (Klein, 1973), (male and immature stages unknown) collected in Stung Chral, Kompong Sela province; *Verrallina* (*Neomacleaya*) *komponga* (Klein, 1973), known only by the male, collected in Kiriom hills, from Kompong Speu province; *Culex* (*Eumelanomyia*) *bokorensis* Klein & Sirivanakarn, 1970, known only by the male, collected in Bokor Hill, in Kampot province and *Mimomyia* (*Ingramia*) *kiriromi* (Klein, 1969), known only by the adults, collected in Kirirom hills, from Kompong Speu province. Our identifications, based on morphological features of adults, focused mainly on female-determination keys; therefore, it is possible that these species (two of them being known only by the male) remained undetected by our prospections. It is difficult to determine whether these species are endemic from Cambodia, or are only the result of a lack of prospection in neighboring countries. Most likely, as they are present in the lowland region of the Greater Mekong Region, they might be present in eastern Thailand or southern Vietnam.

Similarly, some species previously known only from Thailand were collected for the first time in another country [64]. This is the case for *Uranotaenia* (*Pseudoficalbia*) *albipes* Peyton, 1977, which was known only by females in Khao Yai National park in the center of Thailand, that was collected in Rattanak Kiri province in eastern Cambodia. Similarly, *Orthopodomyia* (*Orthopodomyia*) *siamensis* Zavortink, 1968, known only from Muang Trang, was collected in two provinces in eastern Cambodia (Mondul Kiri and Rattanak Kiri). Finally, *Aedes* (*Bothaella*) *eldridgei* Reinert, 1973, collected in Ban Tham Kraeb, Amphoe Chiang Dao in Chiang Mai provinces (North of Thailand), was collected in Mondul Kiri province.

### Comparison with neighboring countries

Being part of the Mekong Sub-region, Cambodia shares ecosystem affinities with its neighbours, especially Thailand, Vietnam and Laos. Out of the 290 species present in Cambodia, 275 species are also present in Thailand, 176 in Vietnam, 75 in Laos, and 18 are considered cosmopolitan species. Using the Sørensen similarity index the Cambodian Culicidae fauna is related at 72.58% with Vietnam, 71.80% with Thailand, and 32.33% with Laos.

The similarity with the Vietnamese and Thai fauna is the strongest. For Vietnam, it can be explained by two factors: first by the continuity of the Tonle Sap Mekong peat swamp forest ecosystem in the south, the Southern Annamite mountain rainforest, and Southeastern Indochina dry evergreen forest ecosystems on the east and the north [109] and secondly, by the low species number in the country. Out of the 191 known species in Vietnam [10], 176 are also known in Cambodia. Vietnam covers more than 331,000 km^2^ and stretches across 1650 km from North to South, and hosts a large range of biologically diverse habitats, most of which have been undersampled. Consequently more species are expected to be found in the country [10]. Further studies in Vietnam are required to assess the actual number of mosquito species [10] and will help to provide a better assessment of the similarities of the two fauna.

Regarding the Thai fauna (with which Cambodia shares 71.80% of its Culicidae fauna), the proximity can be explained by the ecosystem continuity between the two countries, sharing a similar floral pattern (as for instance the Central Indochina dry forests and the Southeastern Indochina evergreen forests). In addition, the eastern part of Thailand belongs to the *lowlands* of the Mekong Region [109], extending through Cambodia, to southern Vietnam, allowing species move across the area.

Finally, the low proximity (32.33%) with Laos can be directly explained by the lack of studies of their Culicidae fauna, having only 170 species [46]. Like for Vietnam, is it most likely that this number is highly underestimated considering the important number of biologically diverse ecosystems in the country. Moreover, the provinces of Rattanak Kiri and Mondul Kiri directly bordering Laos were not investigated in-depth, therefore new records and perhaps new species are expected to be found in this region, and might increase the similarity index percentage with Laos.

### Medically important species

In Cambodia, at least 43 mosquito species are considered to be potentially of medical importance to humans (Table 1).

**Table 1:** List of medically important Culicidae species in Cambodia.

Species	Disease/parasite potentially hosted
*Aedes* (*Aedimorphus*) *vexans*	CHYKV, ZIKAV, JEV, RVFV
*Aedes* (*Ochlerotatus*) *vigilax*	JEV, FILDI, FILWB
*Aedes* (*Stegomyia*) *aegypti*	DENV, ZIKV, CHYKV, RVFV, WNV, YFV
*Aedes* (*Stegomyia*) *albopictus*	DENV, ZIKV, CHYKV, JEV, RVFV, WNV, YFV
*Aedes (Stegomyia) malayensis*	DENV
*Aedes* (*Stegomyia*) *scutellaris*	DENV, YFV
*Aedes* (*Tanakaius*) *togoi*	FILBM, FILWB, FILDI
*Anopheles* (*Anopheles*) *barbirostris*	MAL
*Anopheles (Anopheles) barbumbrosus*	MAL
*Anopheles* (*Anopheles*) *campestris*	MAL
*Anopheles* (*Anopheles*) *donaldi*	MAL
*Anopheles* (*Anopheles*) *lesteri*	MAL
*Anopheles* (*Anopheles*) *letifer*	MAL
*Anopheles* (*Anopheles*) *nigerrimus*	MAL
*Anopheles* (*Anopheles*) *sinensis*	MAL, FILBM
*Anopheles* (*Anopheles*) *whartoni*	FILWB
*Anopheles* (*Cellia*) *aconitus*	MAL
*Anopheles* (*Cellia*) *annularis*	MAL
*Anopheles* (*Cellia*) *baimaii*	MAL
*Anopheles* (*Cellia*) *culicifacies*	MAL
*Anopheles* (*Cellia*) *dirus*	MAL
*Anopheles* (*Cellia*) *epiroticus*	MAL
*Anopheles* (*Cellia*) *indefinitus*	MAL
*Anopheles* (*Cellia*) *jeyporiensis*	MAL, FILBM, FILWB
*Anopheles* (*Cellia*) *karwari*	MAL
*Anopheles* (*Cellia*) *kochi*	MAL
*Anopheles* (*Cellia*) *maculatus*	MAL, FILBM
*Anopheles* (*Cellia*) *minimus*	MAL
*Anopheles* (*Cellia*) *nivipes*	MAL
*Anopheles* (*Cellia*) *philippinensis*	MAL
*Anopheles* (*Cellia*) *vagus*	MAL, JEV
*Armigeres* (*Armigeres*) *subalbatus*	DENV, JEV, FILWB
*Culex* (*Culex*) *fuscocephala*	JEV
*Culex* (*Culex*) *gelidus*	JEV
*Culex* (*Culex*) *quinquefasciatus*	ZIKAV, JEV, RVFV, WNV
*Culex* (*Culex*) *pseudovishnui*	JEV
*Culex* (*Culex*) *sitiens*	JEV, FILBM
*Culex* (*Culex*) *tritaeniorhynchus*	JEV, RVFV
*Culex (Culex) vishnui*	JEV
*Culex* (*Oculeomyia*) *bitaeniorhynchus*	JEV, RVFV, FILBM, FILWB
*Mansonia* (*Mansonioides*) *annulifera*	CHYKV, FILBM
*Mansonia (Mansonioides) uniformis*	RVFV, WNV
*Verrallina* (*Verrallina*) *butleri*	FILDI

DENV = Dengue virus, ZIKV = Zika virus, CHIKV = Chikungunya virus, JEV = Japanese Encephalitis virus, RVFV = Rift Valley Fever virus, WNV = West Nile Virus, YFV = Yellow Fever virus, MAL = *Plasmodium spp*., FILBM = Filariosis from *Brugia malayi*, FILWB = Filariosis from *Wuchereria bancrofti*, FILDI = Filariosis from *Dirofilaria immitis.*

Regarding malaria, 9 species are known to be primary vectors: *Anopheles baimaii, An. barbumbrosus, An. campestris, An. dirus, An. jeyporiensis, An. minimus, An. maculatus, An. philippinensis,* and *An. sinensis.* In all, 13 species are probable – or secondary – vectors, namely: *An. aconitus, An. annularis*, *An. barbirostris, An. culicifacies, An. donaldi, An. epiroticus, An. indefinitus, An. karwari, An. kochi, An. lesteri, An. letifer, An. nigerrimus,* and *An. nivipes* [27, 70, 78, 88, 89, 94].

For arboviruses, regarding dengue, the two main vectors are present: *Ae. aegypti* and *Ae. albopictus.* Three lesser vectors are also recorded: *Ae. scutellaris*, *Ae. malayensis* and *Ar. subalbatus* [28]. Three *Aedes* species are known to be involved in the transmission or able to transmit experimentally, or naturally, yellow fever virus: *Ae. albopictus, Ae. aegypti* and *Ae. scutellaris,* even though no outbreaks have yet been recorded in Asia [8, 13, 106]. The four species *Ae. aegypti, Ae. albopictus, Cx. quinquefasciatus* and *Ma. uniformis* are known to be vectors of the West Nile Fever [98]. The two main vectors of chikungunya*, Ae. albopictus* and *Ae. Aegypti*, are present in Cambodia, along with *Ae. vexans* and *Ma. annulifera* known to be its lesser vectors [6, 28, 95, 104]. Cambodian mosquitoes, especially forest mosquitoes, are most likely involved in the transmission of undiscovered forest arboviruses to wild vertebrate species.

Fourteen species in Cambodia are known to transmit Japanese Encephalitis Virus. The species implicated are *Ae. albopictus, Ae. vexans*, *Ar*. *subalbatus*, *Cx. bitaeniorhynchus, Cx. fuscocephala, Cx. gelidus, Cx. quinquefasciatus, Cx. sitiens, Cx. tritaeniorhynchus,* and *Cx. vishnui* [5]. However, 2 other species are known to be potential vectors: *Ae. vigilax*, and *Cx. pseudovishnui* [5].

In Cambodia, 10 species are known to be potential vectors of filariosis although these parasites have not yet been detected in the country. *Anopheles sinensis*, *An. maculatus*, *Cx. sitiens* and *Ma. annulifera* are known to only transmit *B. malayi*, while *An. jeyporiensis*, *Ae. togoi* and *Cx. bitaeniorhynchus* transmit it along with *W. bancrofti*. *Anopheles whartoni* and *Ar. subalbatus* carry only *W. bancrofti*. Only three species in Cambodia, *Ae. togoi*, *Ae. vigilax* and *Ve. butleri*, can transmit *Dirofilaria immitis* [24, 94].

## Conclusion

A total of 290 mosquito species belonging to 20 genera are recorded from Cambodia with at least 43 medically important species. Forty-nine species are new records for the country. While being only preliminary, this number is expected to increase in the near future, as studies of the Cambodian fauna will continue, including use of genetic/molecular approaches. As many areas of biological interest have not been yet sampled, it is most likely that many new species are to be discovered in remote areas. Further particular efforts should be done on larval prospection and investigation of unusual habitats such as caves, remote forests or karstic massifs. Implementation of new determination techniques, such as MALDI-TOF which focus on the protein profile of the sample, will help to determine specimens rapidly and at lower cost, fastening the identification rate of the entomologists and potentially highlighting cryptic or problematic species.

The influence of climate change will, in the near future, affect the distribution of the mosquitoes and some species might occur in areas where they were previously absent. Similarly, the high deforestation rate the country is actually facing might place in contact mosquitoes vectors, humans and new viruses. This can therefore represent a cradle for the emergence of new infectious diseases. This perspective stresses the essential role of closely monitoring mosquito diversity and the viruses they can carry, in order to defuse any potential threats as much as possible before they appear.
